# Simulation‐Informed Evaluation of Microvascular Parameter Mapping for Diffusion MR Imaging of Solid Tumours

**DOI:** 10.1002/mrm.70318

**Published:** 2026-03-07

**Authors:** Anna Kira Voronova, Olivia Prior, Athanasios Grigoriou, Francesc Salvà, Elena Elez, Luz M. Atlagich, Roser Sala‐Llonch, Marco Palombo, Els Fieremans, Dmitry S. Novikov, Raquel Perez‐Lopez, Francesco Grussu

**Affiliations:** ^1^ Radiomics Group Vall d'Hebron Institute of Oncology (VHIO), Vall d'Hebron Barcelona Hospital Campus Barcelona Spain; ^2^ Department of Biomedicine, Faculty of Medicine and Health Sciences University of Barcelona Barcelona Spain; ^3^ Medical Oncology Service Vall d'Hebron Barcelona Hospital Campus, Vall d'Hebron Institute of Oncology (VHIO) Barcelona Spain; ^4^ Department of Biomedicine, Faculty of Medicine Institut d'Investigacions Biomédiques August Pi i Sunyer (IDIBAPS), University of Barcelona Barcelona Spain; ^5^ Centro de Investigación Biomédica en Red de Bioingeniería, Biomateriales y Nanomedicina Barcelona Spain; ^6^ Cardiff University Brain Research Imaging Centre (CUBRIC), School of Psychology Cardiff University Cardiff UK; ^7^ School of Computer Science and Informatics Cardiff University Cardiff UK; ^8^ Bernard and Irene Schwartz Center for Biomedical Imaging, Department of Radiology New York University Grossman School of Medicine New York NY USA

**Keywords:** cancer, diffusion MRI, microvasculature, modelling, simulations

## Abstract

**Purpose:**

We aim to inform the design of new diffusion MRI (dMRI) approaches for microvasculature quantification that enhance the biological specificity of imaging towards cancer.

**Methods:**

We adopted simulation‐informed modelling of the vascular dMRI signal. We synthesised signals from 1500 synthetic vascular networks, for a variety of protocols (flow‐compensated [FC], non‐compensated [NC], hybrid), featuring different b samplings and diffusion times. We estimated the number of independent, recoverable signal degrees of freedom in presence of noise (signal‐to‐noise ratio of 5), and ranked 12 microvascular metrics depending on the quality of their estimation. Lastly, we demonstrated the feasibility of estimating the top‐ranking metrics on 3T dMRI of a healthy volunteer and of a metastatic colorectal cancer (CRC) patient.

**Results:**

Both NC and FC synthetic vascular signals exhibited complex behaviour as, for example, non‐zero kurtosis and diffusion time dependence. Two independent degrees of freedom appeared recoverable from directionally‐averaged vascular signals (SNR of 5). Mean volumetric flow rate $q_{m}$ and an *Apparent Network Branching* (ANB) index maximised correlations between ground truth and estimated values in silico. In the patient, both $q_{m}$ and ANB detected re‐vascularisation after 3 months of targeted therapy against liver metastases, consistently with *Intra‐Voxel Incoherent Motion* (IVIM) metrics.

**Conclusions:**

Simulation‐based modelling of the vascular dMRI signal suggests $q_{m}$ and ANB as the most promising metrics for tissue microvasculature characterisation. Their estimation in vivo appears feasible to capture general trends, and demonstrates contrasts that are biologically plausible, encouraging their usage in future studies.

## 1 Introduction

Motion‐probing gradients are used in diffusion Magnetic Resonance Imaging (dMRI) to sensitise the MRI signal to different types of spin motion [1], including flow from microperfusion, without relying on contrast agents [2, 3, 4]. One among several proposed approaches for capillary flow quantification based on dMRI is *intra‐voxel incoherent motion* (IVIM) imaging [4, 5, 6, 7], a method for voxel‐wise estimation of the apparent perfusion fraction ($f_{v}$) and of the pseudo‐diffusion coefficient ($D^{*}$) [8, 9]. IVIM indices offer promise in liver malignancy detection [10] or hyper‐vascularisation assessment [11], and correlate with vessel histology [12, 13]. However, despite these encouraging data, IVIM metrics have so far failed to make a lasting impact in the clinic, being semi‐quantitative and protocol‐dependent [14], facts that hinder their clinical adoption.

Recent research has focussed on the design of new signal representations that enhance the biological specificity of dMRI beyond IVIM [4, 8, 9, 15, 16, 17, 18]. In this context, the numerical simulation of blood flow within synthetic vascular networks has shown promise as a way to increase the realism of vascular dMRI signal models, potentially paving the way to a new generation of techniques [19, 20, 21, 22, 23]. *In this work, we adopt this powerful approach and use simulation‐based modelling to inform the development of new dMRI microvascular parameter mapping approaches*.

We used capillary networks traced on histological liver tissue to simulate vascular dMRI signals for realistic flow‐compensated (FC) and non‐compensated (NC) acquisitions [24]. We analysed such signals to compute the number of recoverable, independent vascular parameters at clinical signal‐to‐noise ratio (SNR), and investigated which parameters can be practically retrieved through model fitting. Lastly, we tested the estimation of the most promising microvascular properties in vivo.

## 2 Methods

### 2.1 Vascular Signal Analysis in Silico

#### 2.1.1 Microvascular Networks

We used 15 freely available 2D vascular networks from a recent study [23], derived from liver biopsies (permanent address: https://github.com/radiomicsgroup/SpinFlowSim/tree/main/networks). The networks are characterised by a set of nodes, connected through capillary segments, and feature one inlet and one outlet. We generated 1500 unique networks through 100 three‐dimensional realisations of each 2D network (Supporting Information: Figure S1). For each realisation, we perturbed the (x,y) position of each node, varied each segment diameter, and changed inlet/outlet. Additionally, we also simulated 3D depth using an exponential function, with maximum depth achieved in the network centre. Radii were perturbed of up to ±40% from the original value; x/y node positions of up to ±2μm; and the network depth of $\pm z_{max}=150\mu$m. Moreover, for each perturbation we also removed 3% of the capillary segments. All perturbations were drawn from the uniform distribution. The perturbations were designed to obtain roughly uniform distributions of capillary lengths and radii given the initial set of discrete length and radii. The perturbation depth (Δz=300μm) was chosen to be comparable to the in‐plane network size [23] (ranging from 240 and 600μm). The 3D networks and the code for their generation will be released at the permanent address: https://github.com/radiomicsgroup/SpinFlowSim/tree/main/networks3D.

We simulated an input volumetric flow rate (VFR) $q_{in}$ in 10 equally‐distributed values in [$1.5\cdot 10^{-4}$; $2.75\cdot 10^{-3}$] 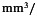
s, and obtained per‐segment VFR q and mean velocity vector v. The range of $q_{in}$ was chosen to generate biologically plausible blood velocity distributions [25], and is in line with previous simulations [23].

Each network instantiation was seeded uniformly with 5000 spins (a trade‐off between simulation accuracy and computational time [23]). The n‐th spin trajectory ${\text{p}}_{n}(t)$ was obtained as

(1) $${\text{p}}_{n}(t)={\text{p}}_{n}(0)+\int_{0}^{t}{\text{v}}_{n}(\xi )\;d\xi ,$$

where ${\text{v}}_{n}(t)$ is the instantaneous spin velocity. Spins experience plug flow, and the velocity field v is derived by solving a pipe network (electric‐hydraulic analogy [23]). At bifurcations, spins are assigned randomly to a branch with a probability proportional to the through‐branch VFR [23]. Simulations were based on the SpinFlowSim flow simulator [23] (https://github.com/radiomicsgroup/SpinFlowSim).

We characterised each network realisation by computing a wide range of properties, namely: statistics of the distributions of the VFR q, velocity v, radius r, and capillary length L; indices describing its size, connectivity and complexity.

Regarding v and q, we computed mean and standard deviation across all capillary segments ($v_{m}$ and $v_{s}$; $q_{m}$ and $q_{s}$). We also computed the path‐weighted mean velocity $v_{w}=E[\frac{\sum_{j}L_{j}v_{j}(L_{j})}{\sum_{j}L_{j}}]$ and the path‐weighted mean VFR $q_{w}=E[\frac{\sum_{j}L_{j}q_{j}(L_{j})}{\sum_{j}L_{j}}]$. In essence, $v_{w}$ (or $q_{w}$) are the line integrals of v (or q) along an input/output (IO) path, normalised by the path length $L^{p}=\sum_{j}L_{j}$, and then averaged over all possible paths (j iterates over the segments making up a single path, while E[·] is the expectation across paths).

We obtained similar indices of mean and path‐weighted mean for the capillary radius ($r_{m}$ and $r_{w}$) and also computed (i) the mean capillary segment length $L_{m}$, (ii) the mean IO path length $L_{m}^{p}=E[L^{p}]$, (iii) the number of IO paths $N_{paths}$, and (iv) the *apparent network branching* (ANB) [23]. ANB estimates the average number of capillary segments spins travel through during a reference time of 100 ms. Such a long reference time ensures that flowing spins have sufficient time to explore the network topology.

#### 2.1.2 dMRI Signal Synthesis

Motion‐sensitising gradient waveforms G(t) were used to obtain magnitude dMRI signals as [26]

(2) $$s=\left|\frac{1}{N}\overset{N}{\underset{n=1}{\sum }}e^{\;-i\;\gamma \;\int_{0}^{TE}p_{n}(t)\;\cdot \;G(t)\;dt}\right|,$$

where N=5000 is the number of spins, n the spin index, $p_{n}(t)$ the n‐th spin trajectory and TE the echo time. Equations (1) and (2) where discretised for their practical numerical implementation, using a temporal resolution of Δt = 10 μs.

We used NC pulsed‐gradient spin echo (PGSE) monopolar waveforms [27] and FC bipolar waveforms [28], compensating for velocity. Both NC and FC gradients were linearly polarised [29], and both refocus stationary spins, since $\int_{0}^{TE}G(t)dt=0$. FC waveforms also refocus spins flowing at constant velocity (ballistic regime [9]), since they null the 1st gradient moment ($\int_{0}^{TE}t\;G(t)dt=0$). NC waveforms were parametrised by the diffusion gradient duration/separation/strength δ/Δ/G, while FC waveforms by G, Δ and by the oscillation half‐period τ (Supporting Information: Figure S2). Note that $b=\gamma^{2}G^{2}\delta^{2}(\Delta -\delta /3)$ for the NC waveforms, and $b=\frac{4}{3}\gamma^{2}G^{2}\tau^{2}$ for the FC ones. The diffusion time is Δ−δ/3 for the NC protocol and τ for the FC one.

We synthesised signals for several different protocols, using 15 uniformly‐distributed gradient directions [30] for each b. The protocols were:

- A *NC protocol*, with b‐values matching the in vivo acquisitions (see below), that is, b={0, 10, 20, 40, 70, 100} s/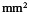. We used a fixed Δ of either 30 or 50 ms, and gradient duration δ=6 ms. Signals from the 15 directions were averaged. This protocol is representative of a standard IVIM acquisition, usually acquired at fixed diffusion time. The effect of changing the diffusion time is also tested by using two different Δ values.
- A *FC protocol*, with the same 6 b‐values, as above. We fixed Δ to 30 ms, and used a half‐period τ of either 3 or 10 ms. Signals from the 15 directions were averaged. This protocol tests whether compensating the signal decay from spins moving at constant velocity offers any advantages compared to standard IVIM‐like monopolar encoding.
- A *hybrid protocol*, obtained by alternating NC and FC b‐values from the two protocols above (same theoretical duration as the FC and NC protocols above). This protocol tests whether combining FC and NC measurements widens the range of signal contrasts probed during a fixed scan time, and hence improves microvascular parameter estimation.
- A *richNC protocol*, featuring NC PGSE measurements as above (Δ=30 ms, δ=6 ms), but a richer b‐value sampling (20 b‐values in the range [0; 100] s/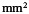). Two versions of the protocol were obtained: one with directional averaging at fixed b, and one without averaging. This protocol tests whether denser b‐samplings provide additional information on the underlying microvascular properties, compared, for example, to the more standard NC protocol above.
- A *richFC protocol*, featuring FC measurements as above (Δ=30 ms, τ=10 ms), but again, a richer b sampling (20 values of b in [0; 100] s/$mm^{2}$), and again with/without directional averaging. This protocol tests the effect of acquiring denser b‐samplings in presence of flow compensation.
- A *hybrid rich protocol*, obtained by alternating richNC and richFC b‐values from the two protocols above (same theoretical duration as the richFC and richNC protocols above). This protocol tests the effect of acquiring denser b‐samplings when both FC and NC contrasts are probed.

Directional averaging was achieved by computing the arithmetic mean of the signal over the 15 diffusion directions.

An equivalent scan time can be calculated for each protocol assuming: (i) an acquisition time of 11 s/image (in line with our in vivo data—see Section 2.2); (ii) a signal averaging factor of 2; (iii) the acquisition of two additional b‐values larger than 100 s/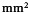 to characterise extra‐vascular tissue signals. This leads to the approximate scan time of: 8 min with 3 directions per b‐value (standard trace imaging) and 40 min with 15 directions per b‐value for each of the NC, FC and hybrid NC‐FC protocols; 115 min for the richNC, richFC and hybrid richNC‐richFC protocol with 15 directions per b‐value. Note that the “rich” protocols are not practically feasible in the radiology clinic, but may be employed in some contexts in the research setting.

We also visualised dMRI signals for a wide range of diffusion times, that is, Δ={10,50,100} ms for both FC and NC protocols (with δ=0.5 ms for the NC protocol and τ=1 ms for the FC one). Note that the FC and NC protocols introduced above (FC, NC, richFC, richNC) probe different time scales due to their considerably different diffusion times: while the minimum diffusion time of the NC protocol is of 28 ms, the maximum diffusion time of the FC protocol is 10 ms, i.e., considerably shorter than that of the NC protocol.

Signals were corrupted with Rician noise (SNR of 5 and 20 at b=0).

#### 2.1.3 Analysis: Degrees of Freedom Estimation

We performed Singular Value Decomposition (SVD) of matrices storing noisy and noise‐free signals to estimate the number of independent, detectable microvascular degrees of freedom $N_{p}$ [31, 32]. We stacked all Q=1500 microvascular signals in matrices of size Q×M, where M is the number of protocol measurements, and computed the min(Q,M) independent SVs. For each protocol and SNR, we compared noisy/noise‐free SVs λ to estimate $N_{p}$ by counting the noisy SVs such that

(3) $$\frac{\left|\lambda^{\text{noisy}}-\lambda^{\mathrm{noise}-\mathrm{free}}\right|}{\lambda^{\mathrm{noise}-\mathrm{free}}}\leq \theta .$$

We computed the threshold θ automatically using Marchenko‐Pastur Principal Component Analysis (MP‐PCA) [31, 32] (python implementation at the permanent address: https://github.com/NYU‐DiffusionMRI/mppca_denoise) in the protocols featuring large M (e.g., rich protocols). In the remaining protocols, which do not contain sufficient data redundancy to enable MP‐PCA [31, 32], we calculated $N_{p}$ by varying θ from 0.07 to 0.80. Note that no pre‐whitening was required for SV computation, since (i) we use the same noise level σ for all synthetic voxels at fixed SNR, and (ii) SV thresholding is a valid strategy to study the number of recoverable signal components even in presence of Rician noise [31].

#### 2.1.4 Analysis: Vascular Property Estimation and Ranking

**FIGURE 1:**
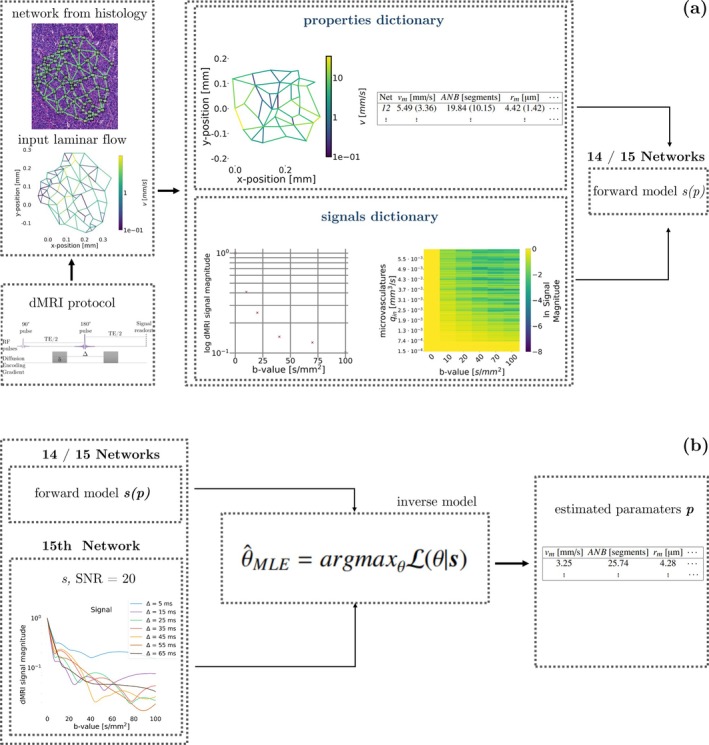
Illustration of the simulation‐informed framework developed to study microvascular parameter inference in silico, which relies on 15 synthetic vascular networks available from a previous study. (a) Firstly, synthetic dMRI signals for several acquisition protocols were generated using *SpinFlowSim*. Noise‐free signals from 14 out of 15 networks in turn were used to build numerical forward models based on radial basis functions (RBFs), predicting dMRI signals from a vascular property of interest. (b) Afterwards, the numerical signal models were plugged into standard maximum‐likelihood fitting routines, which estimated such a vascular property from noisy signals from the 15th network, in a leave‐one‐out fashion. In summary, RBF models were built using 1400 coupled noise‐free signals and vascular parameters, and then deployed on 100 unseen noisy signals. The leave‐one‐network‐out strategies delivers predictions for all 1500 network realisations.

We investigated which microvascular properties can be best inferred from noisy measurements (Figure 1). We trained radial basis function (RBF) [23] numerical forward models on noise‐free signals from 14 out of 15 networks. Afterwards, the trained RBF models were fitted to noisy signals from the 15th network through maximum‐likelihood inference [33], in a leave‐one‐out fashion, estimating each vascular property in turn. Since each network features 100 unique realisations, this implies that each RBF model was built using 1400 coupled noise‐free signals/vascular parameters, and then deployed on 100 unseen noisy signals. In practice, the RBF models were fitted to signal measurements by minimising an objective function defined as the negative of the log‐likelihood ln(L) under an offset Gaussian noise model [33] ($f_{obj}=-\;\ln(L)$), that is,

(4) $$f_{obj}(u,h)=\frac{M}{2}\;\ln(\sqrt{2\pi \sigma^{2}})\;+\;\frac{1}{2\sigma^{2}}\sum_{m=1}^{M}{\left(a_{m}-\sqrt{s_{m}^{2}(u,h)+\sigma^{2}}\right)}^{2}.$$

Above, u is the vector of microvascular parameters to be estimated, h is the vector of hyperparameters of the RBF model, M is the number of measurement, $\sigma^{2}$ is an estimate of the noise variance, while $a_{m}$ and $s_{m}(u,h)$ are the m‐th noisy signal measurement and its corresponding prediction from the RBF model. Note that during RBF model training, the signal measurements $a_{m}$ and the vascular parameters u are fixed, and used to find the optimal hyperparameters h. Afterwards, during the deployment of the trained RBF model on unseen noisy signals, h is kept fixed, enabling the estimation of the microvascular parameters through maximum‐likelihood fitting. In practice, we minimised $f_{obj}$ in Equation (4) with respect to each microvascular parameter at a time (i.e., modelling u=[u] as a scalar).

We compared the estimated and ground truth properties by computing the Spearman's correlation coefficient $r_{s}$, and a Bias Index (BI) [34]. Both were evaluated pooling together predictions for all 1500 networks. Confidence intervals were obtained by recording ranges across leave‐one‐out iterations. Properties were ranked according to decreasing $r_{s}$, thus assessing the sensitivity of the signal to each property and their practical detectability in presence of noise. Once the two most robust microvascular properties were identified through $r_{s}$ ranking, we repeated parameter estimation by inferring these two properties together (i.e., parametrising $f_{obj}$ in Equation (4) as a function of both, with a two‐dimensional parameter vector $u={\left[u_{1},\;u_{2}\right]}^{T}$).

For comparison, we also ranked properties according to increasing values of the BI (in absolute value), to assess the accuracy of parameter estimation, beyond sensitivity and detectability assessments based on $r_{s}$.

#### 2.1.5 Analysis: Relationship Between Vascular Properties and Vascular Signal Features

Finally, we investigated how the top‐ranking metrics are encoded in the vascular dMRI signal, by assessing their relationship with the main dMRI signal cumulants, that is, the apparent pseudo‐diffusion and kurtosis coefficients, referred to as $D^{*}$ and $K^{*}$. These characterise the signal slope and curvature in log‐scale as a function of the b‐value and, to our knowledge, are the most common descriptors used to capture the behaviour of the dMRI signal decay as a function of b. These were computed by fitting

(5) $$s=e^{-b\;D^{*}+\frac{1}{6}\;K^{*}{\left(b\;D^{*}\right)}^{2}}.$$

We computed the Spearman's correlation coefficient $r_{s}$ between the top‐ranking vascular properties and $D^{*}$/$K^{*}$, and between the two top‐ranking metrics themselves. Note that this analysis was performed only for the NC, FC, richNC, and richFC protocols, as $D^{*}$ and $K^{*}$ cannot be interpreted easily when mixing NC and FC encodings, as done in the hybrid protocols.

### 2.2 Vascular Signal Analysis in Vivo

Lastly, we demonstrated the feasibility of estimating the top‐ranking metrics from the in silico study on in vivo images. For this purpose, we scanned a 32‐year‐old male healthy volunteer and a 50‐year‐old male patient suffering from colorectal cancer (CRC), with liver metastases. The patient was scanned immediately before receiving targeted therapy based on Ecorafenib, Binimetinib and Cetuximab, and after 3 months of treatment. Informed written consent was obtained, and the study was approved by the Vall d'Hebron University Hospital Research Ethics Committee (Barcelona, Spain; CEIm PR(AG)362/2021, PR(IDI)109/2022).

#### 2.2.1 MRI Acquisition and Processing

Volunteers were scanned on a 3T GE SIGNA Pioneer scanner at abdominal level. The protocol included structural imaging and dMRI, with salient parameters: resolution $2.4\times 2.4\times 6{\text{mm}}^{3}$, TE = 75 ms, TR = 12 s (respiratory‐gated), bandwidth 3333 Hz/pixel, trace DW imaging; NEX = 2; parallel imaging factor of 2, $b=\{0,10,20,40,70,100,500,1000,1250,1500\}s/{\text{mm}}^{2}$, with gradient timings: δ={0.00,2.06,2.57,3.37,4.18,4.82,11.97,16.15,18.77,21.12} ms; Δ={0.00,31.34,31.85,32.65,33.47,34.10,25.23,29.41,32.03,34.38} ms. The scan time was of approximately 10 min, that is, roughly 11 s per b‐value, gradient direction and signal average. Note that the diffusion time varies slightly across b‐values, and that diffusion times were calculated automatically by the scanner software and could not be controlled directly. In other systems/implementations, the diffusion time may be instead kept constant across b‐values.

The scan was post‐processed with routine pipelines [23], obtaining voxel‐wise estimates of the vascular signal at b≤100 s/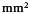. The pipeline included MP‐PCA denoising [32], Gibbs unringing [35] and motion correction [23]. Per‐voxel maps of the top‐ranking microvascular properties from the in silico study were obtained, using numerical models built on all 1500 synthetic networks. Standard IVIM vascular signal fraction and pseudo‐diffusion coefficient ($f_{V}$ and $D^{*}$) were also obtained through segmented fitting [23, 36].

Briefly, segmented fitting consisted in estimating the extra‐vascular (EV) apparent diffusion coefficient $D_{EV}$ on 100 s/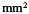 <b<1500 s/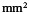 measurements (assuming negligible contributions from the vascular compartment), and then on using the estimated EV parameters to extrapolate the extra‐vascular signal at b≤100 s/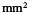 as

(6) $$S_{EV}\approx S_{EV}(b=0)\;e^{-b\;D_{EV}}.$$

Ultimately, the approach enables the estimation of the pure vascular signal $S_{V}$ for b≤100 s/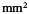 as

(7) $$S_{V}=S\;-\;S_{EV},$$

where S is the measured dMRI signal in a voxel. The normalised signal $S_{V}/S_{V}(b=0)$ can be compared directly to our synthetic vascular dMRI measurements through RBF fitting, for which a new set 1500 synthetic vascular signals was generated. The protocol used for signal synthesis matched exactly all the acquired (b,δ,Δ) values.

#### 2.2.2 Analysis: Vascular Metric Characterisation

We computed mean and standard deviation of all metrics within regions‐of‐interest (ROIs) placed on: liver metastases (patient only); liver parenchyma; spleen. An experienced radiologist (L.M.A.) identified the metastases.

## 3 Results

### 3.1 Vascular Signal Analysis in Silico

#### 3.1.1 Microvascular Networks

Supporting Information: Figure S1 visualises the 2D networks [23] and the 3D network generation procedure.

#### 3.1.2 dMRI Signal Synthesis

**FIGURE 2:**
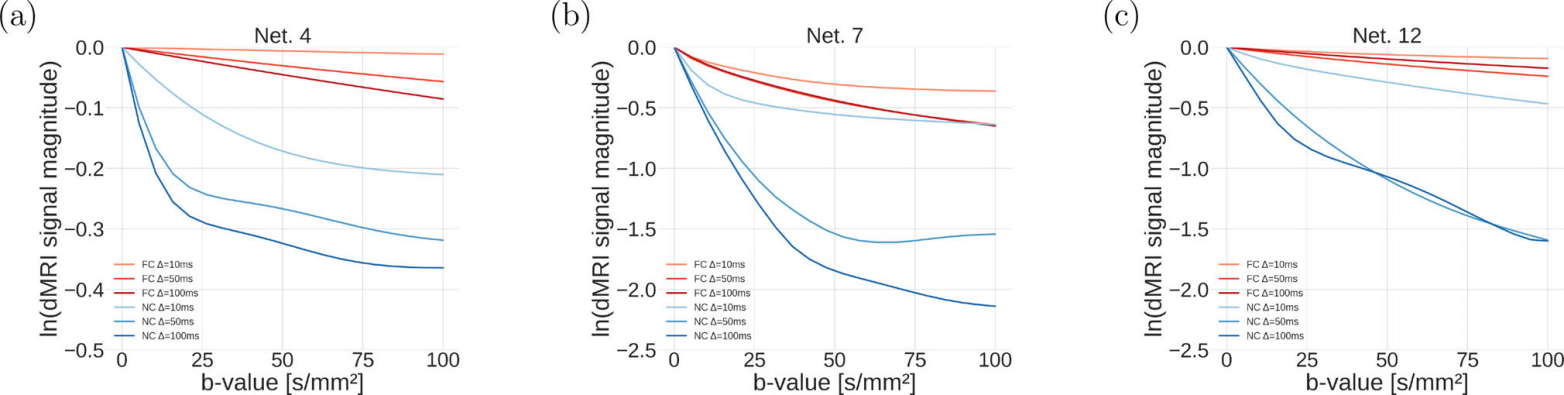
Illustration of the dMRI signal decay as a function of the b‐value for three representative networks ((a): network 4; (b): network 7; (c): network 12). The figure reports decay for both NC and FC gradient waveforms, for different diffusion times. For the NC protocol: δ fixed to δ=0.5 ms, Δ={10,50,100} ms. For the FC protocol: τ fixed to = 1 ms, and Δ={10,50,100} ms. On the y‐axis, we plot the logarithm of the synthetic vascular signal, to better highlight departures from the mono‐exponential signal decay, which would be represented as a straight line. Non‐monoexponential decay is seen for both protocols, as well as a wide range of signal attenuations. The input VFR was $q_{in}=1.6\cdot 10^{-3}$ 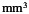/s for all curves.

Figure 2 shows examples of dMRI signal decay as a function of b for three networks (panels (a) to (c)), as Δ varies (one illustrative realisation for networks 4, 7, and 12, following the nomenclature of Supporting Information: Figure S1; input VFR $q_{in}=1.6\cdot 10^{-3}$ 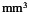/s for all cases). NC protocols are characterised by stronger signal decay than FC protocols. Moreover, the former show a strong dependence on Δ. Non‐monoexponential decay is seen for both, with a wide range of signal attenuations. The synthetic signals are robust to variations in the number of spins used for simulations as long as roughly 3000 spins or more are employed (Supporting Information: Figure S3). Supporting Information: Figure S4 visualises signals for all FC and NC protocols generated for an exemplificatory network (net 7). Signal decay trends are in line with what is observed in Figure 2 (e.g., stronger decay in NC than FC protocols).

#### 3.1.3 Degrees of Freedom Estimation

**FIGURE 3:**
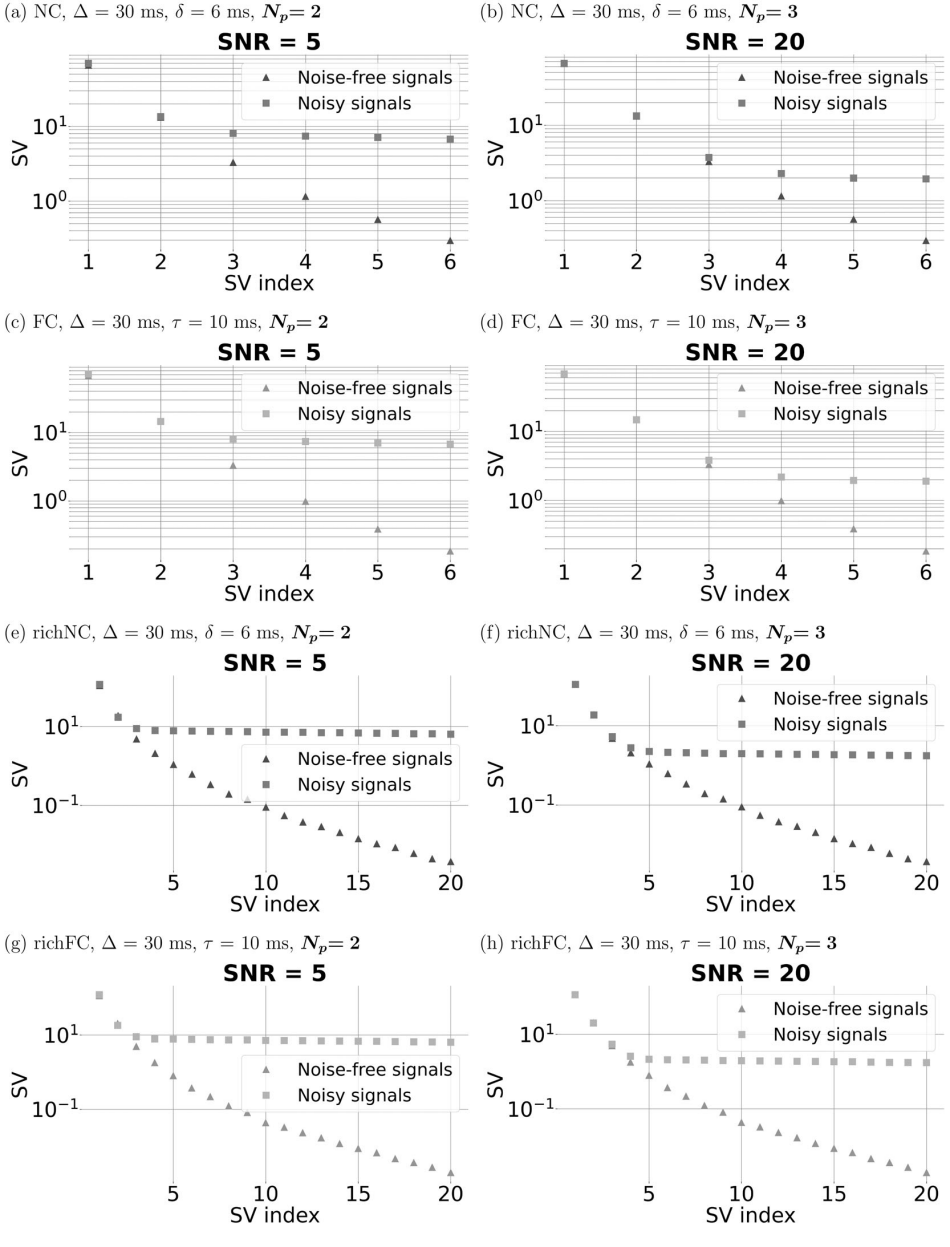
SVD for all non‐compensated (NC) and flow‐compensated (FC) protocols following directional averaging. Left, panels (a), (c), (e), (g): vascular dMRI signal for SNR of 5 at b=0; right, panels (b), (d), (f), (h): vascular dMRI signal for SNR of 20 at b = 0. From top to bottom: NC protocol ((a) and (b)); FC protocol ((c) and (d)); richNC protocol ((e) and (f)); richFC protocol ((g) and (h)). The figure refers to the fixed diffusion time of δ=6 ms and Δ=30 ms (protocols NC and richNC), and of τ=10 ms and Δ=30 ms (protocols FC and richFC).

Figure 3 reports SVD for directionally‐averaged signals for all protocols. The figure refers to the fixed diffusion time of δ=6 ms and Δ=30 ms for NC and richNC protocols; of τ=10 ms and Δ=30 ms for FC and richFC protocols. $N_{p}=2$ independent SVs are detectable for SNR = 5 for all protocols, and $N_{p}=3$ for SNR = 20. Note that the figure refers to the case when a SV threshold of θ=0.17 is used. These findings are confirmed for different values of θ (Supporting Information: Table S1).

Supporting Information: Figure S5 shows similar results for a different diffusion time (δ=6 ms and Δ=50 ms for the NC protocol; τ=3 ms and Δ=30 ms for the FC protocol). Results are in line with those of Figure 3.

**FIGURE 4:**
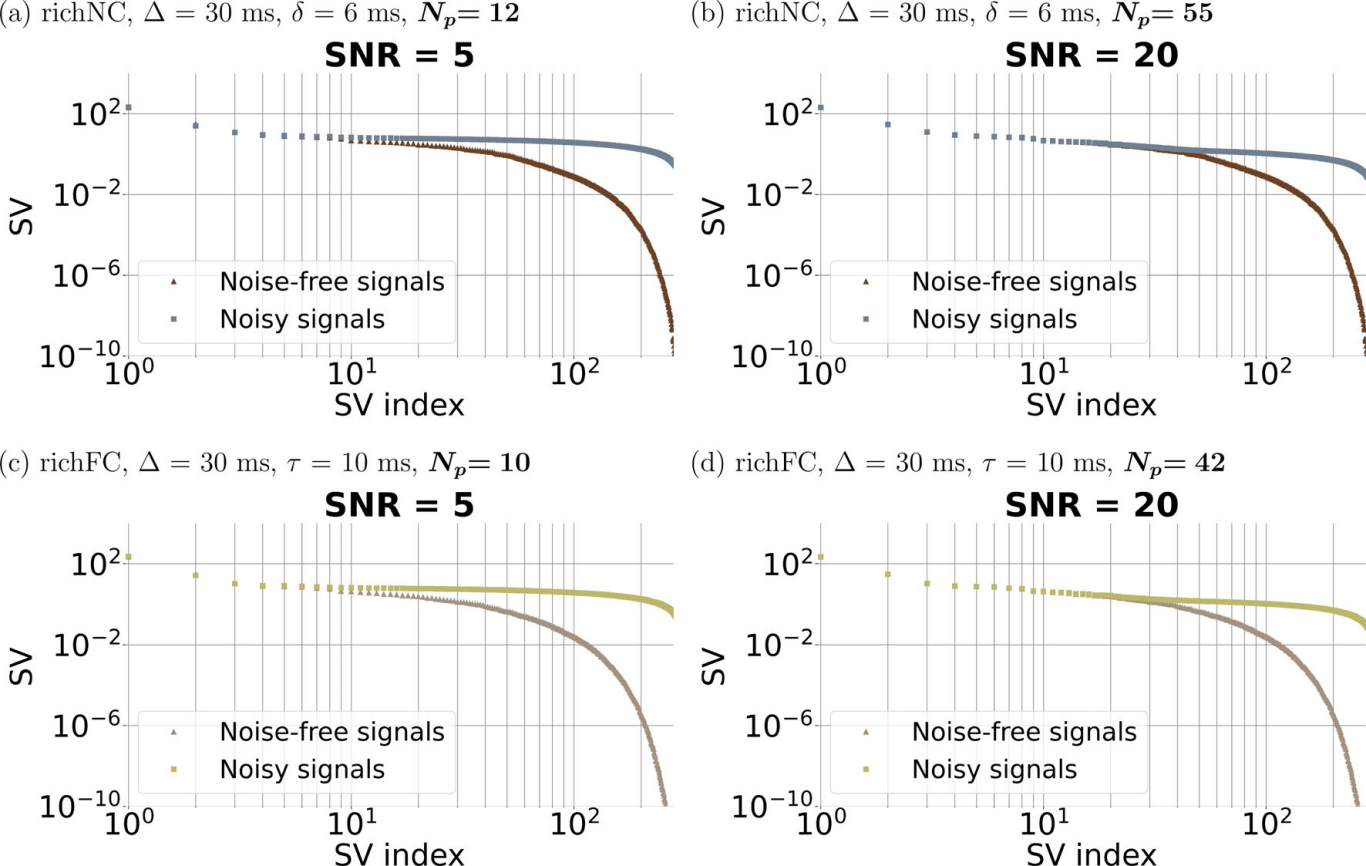
SVD for the rich non‐compensated (richNC) and rich flow‐compensated (richFC) protocols without directional averaging. Left, panels (a), (c): vascular dMRI signal for SNR of 5 at b=0; right, panels (b), (d): vascular dMRI signal for SNR of 20 at b=0. From top to bottom: richNC protocol ((a) and (b)); richFC protocol ((c) and (d)). The figure refers to the fixed diffusion time of δ=6 ms and Δ=30 ms (richNC), and of τ=10 ms and Δ=30 ms (richFC).

Figure 4 shows SVs for the rich protocols when directional averaging is not performed. SVD yields a considerably higher number of independent SVs: we detect $N_{p}=10$ (richFC protocol) and $N_{p}=12$ (richNC protocol) for SNR = 5, and $N_{p}=42$ (richFC protocol) and $N_{p}=55$ (richNC protocol) for SNR = 20.

#### 3.1.4 Vascular Property Estimation and Ranking

**FIGURE 5:**
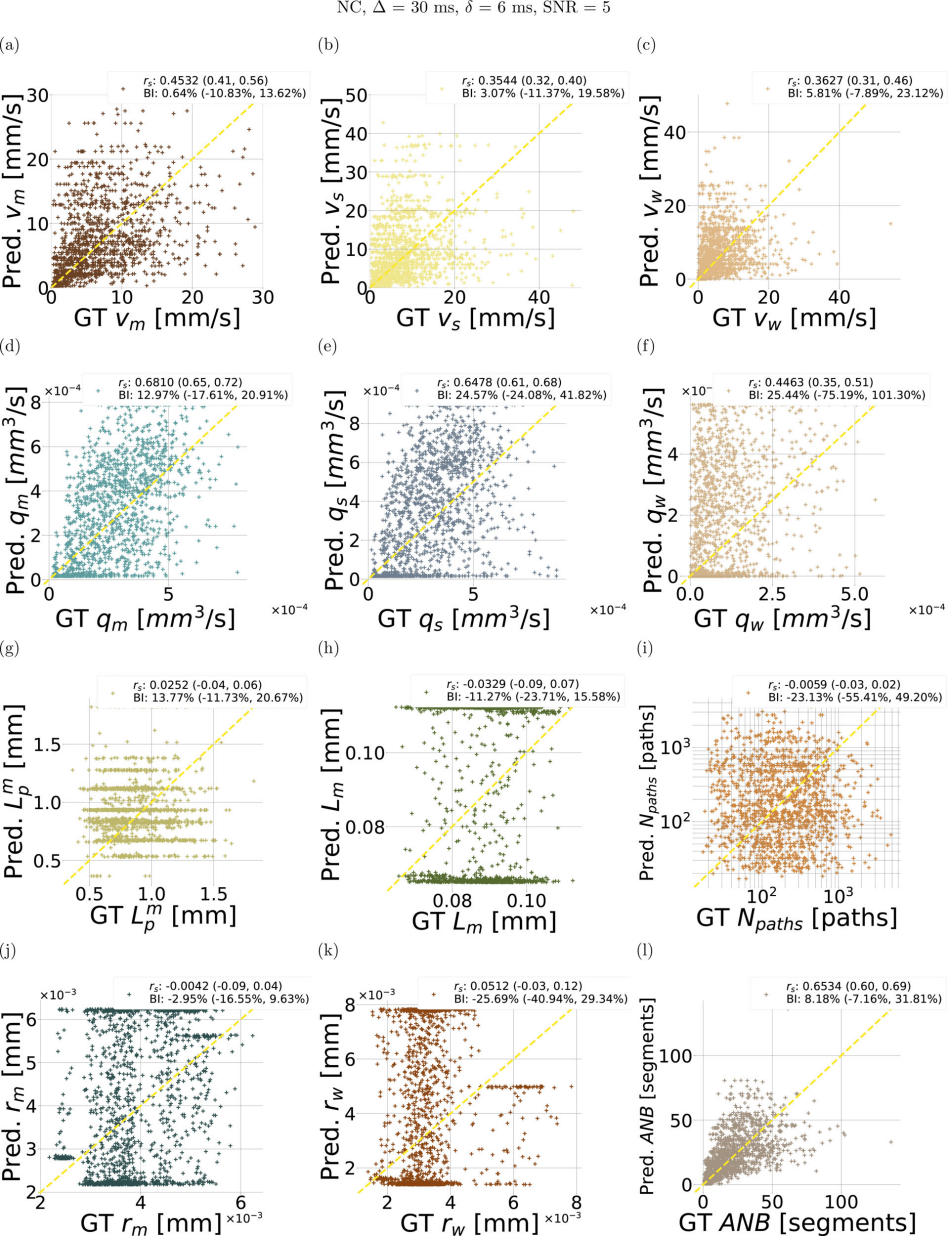
Scatter plots of estimated vascular parameters against ground truth values from the leave‐one‐out fitting procedure implemented in silico. The figure refers to the NC protocol, with Δ=30 ms and δ=6 ms, SNR = 5. From top to bottom: first row, mean velocity $v_{m}$ in (a), standard deviation of velocity $v_{s}$ in (b), path‐weighted mean velocity $v_{w}$ in (c); second row, mean volumetric flow rate (VFR) $q_{m}$ in (d), standard deviation of VFR $q_{s}$ in (e), path‐weighted mean VFR $q_{w}$ in (f); third row, mean input/output path length $L_{p}^{m}$ in (g), mean capillary segment length $L_{m}$ in (h), number of input/output paths $N_{paths}$ in (i); fourth row, mean capillary radius $r_{m}$ in (j), path‐weighted mean capillary radius $r_{w}$ in (k), and apparent network branching ANB in (l). For each metric, the overall Spearman's correlation coefficient $r_{s}$ and Bias Index (BI) are reported, with the range of $r_{s}$ and BI values obtained across leave‐one‐out folds. “GT” and “Pred.” respectively indicate ground truth and predicted metric values.

Figure 5 shows scatter plots comparing ground truth against predicted vascular parameters for the NC protocol (Δ=30 ms, δ=6 ms, SNR of 5). The figure also reports Spearman's correlation coefficients $r_{s}$ and the BI for all metrics. Supporting Information: Figure S6 reports similar results for the FC protocol (Δ=30 ms, τ=10 ms). In both figures, the quality of the estimation varies greatly across parameters. Estimates of velocity and VFR distribution moments (e.g., $v_{m}$ and $q_{m}$) closely agree with ground truth values, unlike metrics related to the capillary geometry, which cannot be estimated (e.g., $r_{s}$ of 0.681 for $q_{m}$ against 0.025 for $L_{p}^{m}$). Strong correlations with ground truth and limited bias (BI < 8 %) are achieved for ANB in both protocols. Higher values of $r_{s}$ are observed for the FC than the NC protocol (e.g., $r_{s}$ of 0.453, 0.681, and 0.653 for $v_{m}$, $q_{m}$ and ANB for NC, while of 0.496, 0.733, 0.668 for the FC).

Supporting Information: Figures S7 and S8 show scatter plots for SNR = 20. The estimation of metrics such as $v_{m}$, $q_{m}$ and ANB benefits from the higher SNR, while geometry parameters $L_{m}$, $L_{p}^{m}$, $N_{paths}$, $r_{m}$ and $r_{w}$ are still estimated poorly. Supporting Information: Figures S9 and S10 show estimation results for Δ=50 ms, δ=6 ms (NC protocol) and Δ=30 ms, τ=3 ms (FC protocol). Estimation performances improve slightly as the diffusion time increases. The correlation between ground truth and estimated mean VFR $q_{m}$ is consistently higher than figures obtained for mean blood velocity $v_{m}$ across all acquisition configurations and noise levels.

**FIGURE 6:**
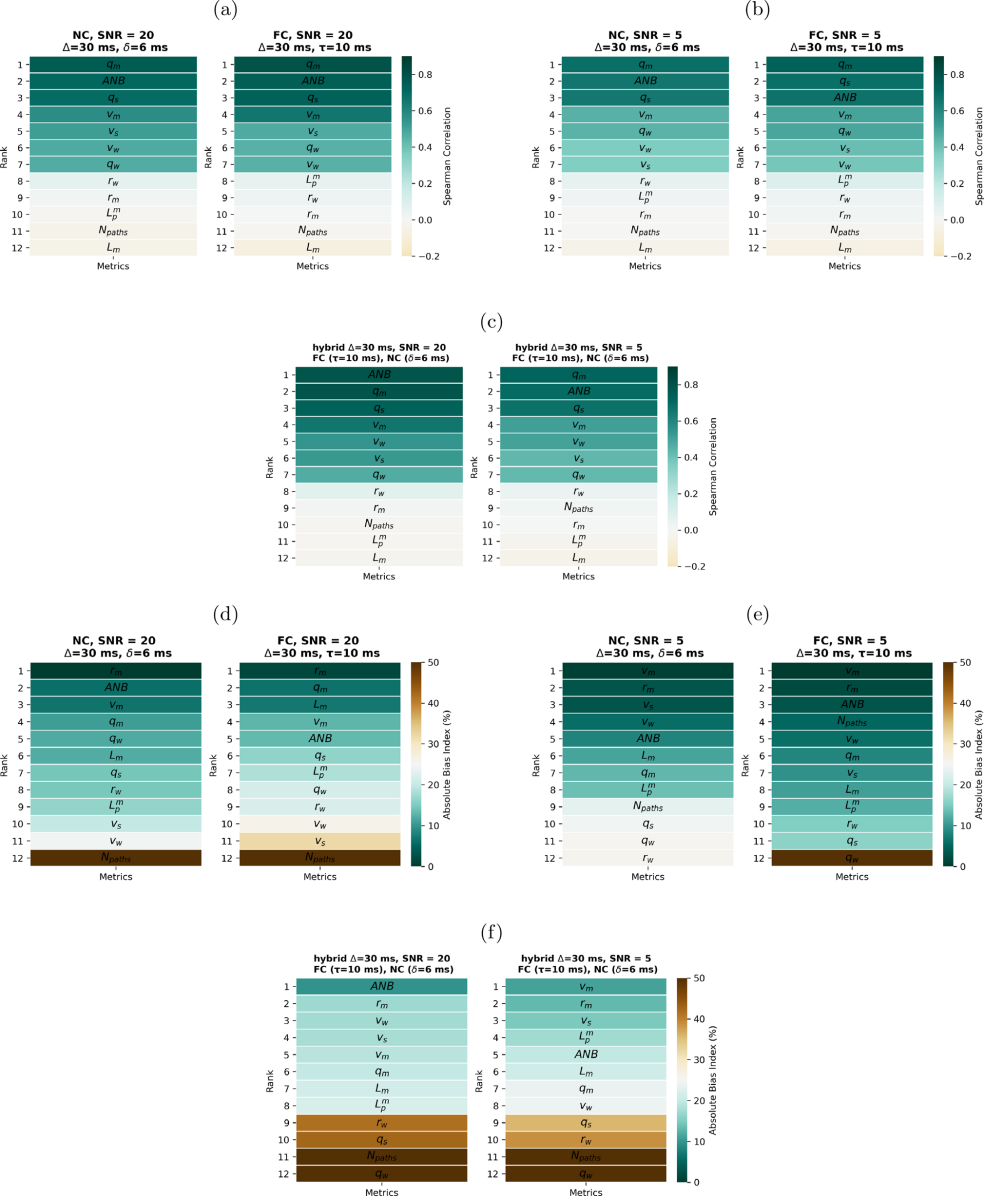
Ranking of microvascular metrics based on the Spearman's correlation coefficient ($r_{s}$) and on the absolute value of the Bias Index (BI) between ground truth and estimated microvascular metric values. Ranking was obtained for the NC and FC protocols for SNR = 20 and SNR = 5, and for a hybrid protocol alternating measurements from the NC and the FC gradient waveforms. (a) $r_{s}$ ranking for the NC and FC protocols (SNR = 20); (b) $r_{s}$ ranking for the FC and NC protocol (SNR = 5); (c) $r_{s}$ ranking for the hybrid protocol (SNR = 20 and SNR = 5); (d) BI ranking for the NC and FC protocols (SNR = 20); (e): BI ranking for the FC and NC protocol (SNR = 5); (f): BI ranking for the hybrid protocol (SNR = 20 and SNR = 5). The figure reports results for Δ=30 ms, δ=6 ms for the NC and hybrid protocol; Δ=30 ms, τ=10 ms for the FC and hybrid protocol.

Figure 6 and Supporting Information: Figure S11 show rankings based on $r_{s}$ and BI for the NC, FC, and hybrid protocols. Similar rankings are also reported for the rich protocols in Supporting Information: Figure S12. In all cases, $q_{m}$, $q_{s}$, and ANB are the top‐ranking metrics according to $r_{s}$, albeit with slightly different orders depending on the protocol and SNR. The adoption of a hybrid FC/NC acquisition strategy positively impacts on the ranking figures, especially at low SNR, where the estimation of $q_{m}$, ANB, and $q_{s}$ outperforms FC and NC protocols. When BI rankings are considered instead, results are more variable and $q_{m}$ and ANB are not necessarily the top‐ranking metrics. For example, for SNR = 5, statistics of the blood velocity distributions such as $v_{m}$ and $v_{w}$ rank higher on BI than $q_{m}$ for both NC protocol (at Δ=30 ms and again at Δ=50 ms) and FC protocol (at τ=10 ms and again at τ=5 ms).

Supporting Information: Figure S13 shows a final example in which $q_{m}$ and ANB are estimated jointly, with performances comparable to those obtained when the metrics are estimated individually (all cases above). We remark the in Figure 5 and Supporting Information: Figures S6 to S10, vascular parameters were estimated one at a time, from the same noisy MRI signals.

*Given these consistent trends, in the following material we will focus on the estimation of VFR distribution moments and of* ANB. *When considering two tissue parameters, we will focus on* $q_{m}$ *(first VFR moment) and on* ANB.

#### 3.1.5 Relationship Between Vascular Properties and Vascular Signal Features

**FIGURE 7:**
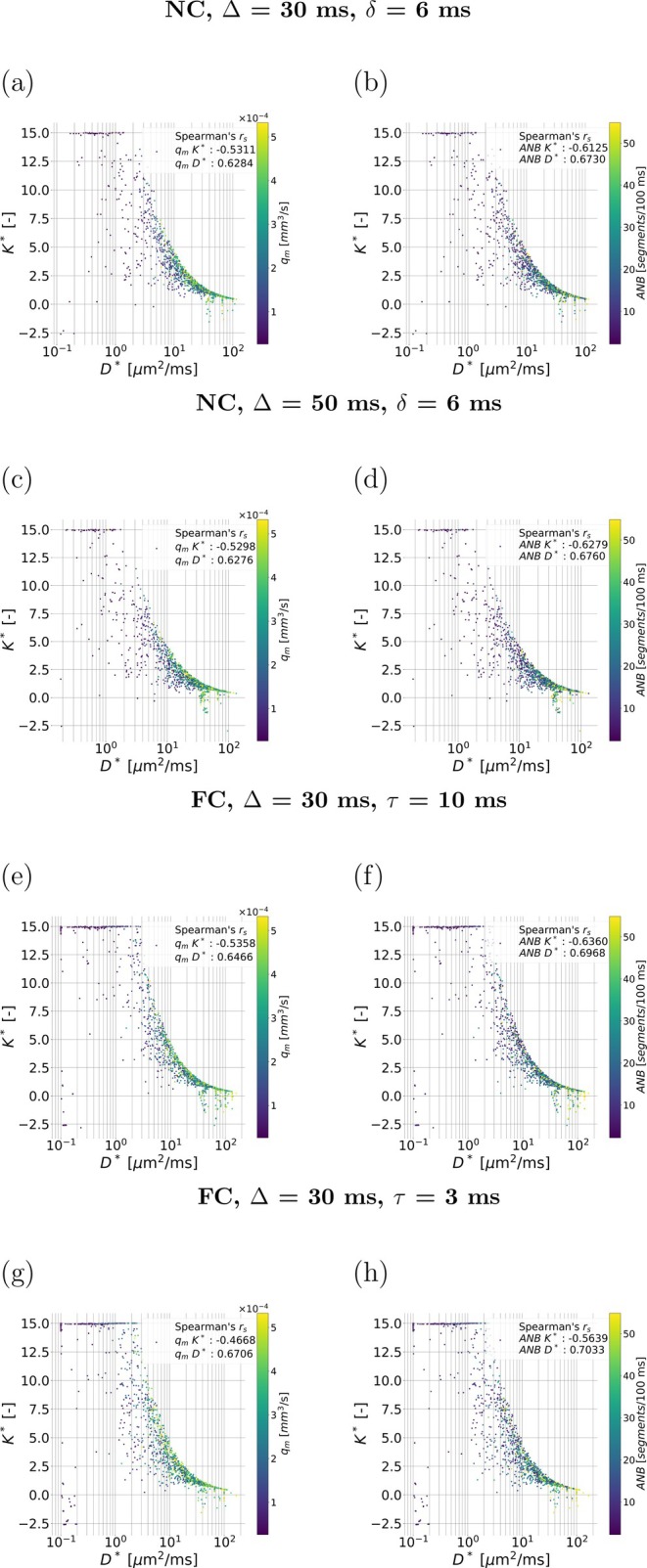
Scatter plots visualising the relationship between the vascular dMR signal cumulants and the top‐ranking metrics selected from the in silico study. The figure scatters the apparent vascular diffusion and kurtosis coefficients ($D^{*}$ and $K^{*}$) against each other, colouring the points according to the mean VFR $q_{m}$ and the apparent network branching ANB. Panels (a) and (b), top row: NC protocol, Δ=30 ms, δ=6 ms ($q_{m}=f(D^{*},K^{*})$ in (a); $ANB=f(D^{*},K^{*})$ in (b)). Panels (c) and (d), second row: as (a) and (b), but for Δ=50 ms, δ=6 ms. Panels (e) and (f), third row: FC protocol, Δ=30 ms, τ=10 ms ($q_{m}=f(D^{*},K^{*})$ in (e); $ANB=f(D^{*},K^{*})$ in (f)). Panels (g) and (h), fourth row: as (e) and (f), but for Δ=50 ms, τ=3 ms. Spearman's correlation coefficients between $q_{m}$ and $D^{*}$ and $K^{*}$, and between ANB and $D^{*}$ and $K^{*}$, are also reported.

Figure 7 shows the dependence of metrics $q_{m}$ and ANB on $D^{*}$ and $K^{*}$. There is a moderate‐to‐strong association between $D^{*}$, $K^{*}$ and either of $q_{m}$ and ANB. The correlation strength is similar for both diffusion times for a given protocol and between FC and NC protocols. $D^{*}$ increases for increasing diffusion time.

Supporting Information: Figure S14 includes similar plots for the rich protocols. The same trends are seen. Additionally, the figure highlights that while $q_{m}$ and ANB are positively correlated ($r_{s}$ of 0.718), a range of ANB values can be observed for any given $q_{m}$.

### 3.2 Vascular Signal Analysis in Vivo

#### 3.2.1 Vascular Metric Characterisation

**FIGURE 8:**
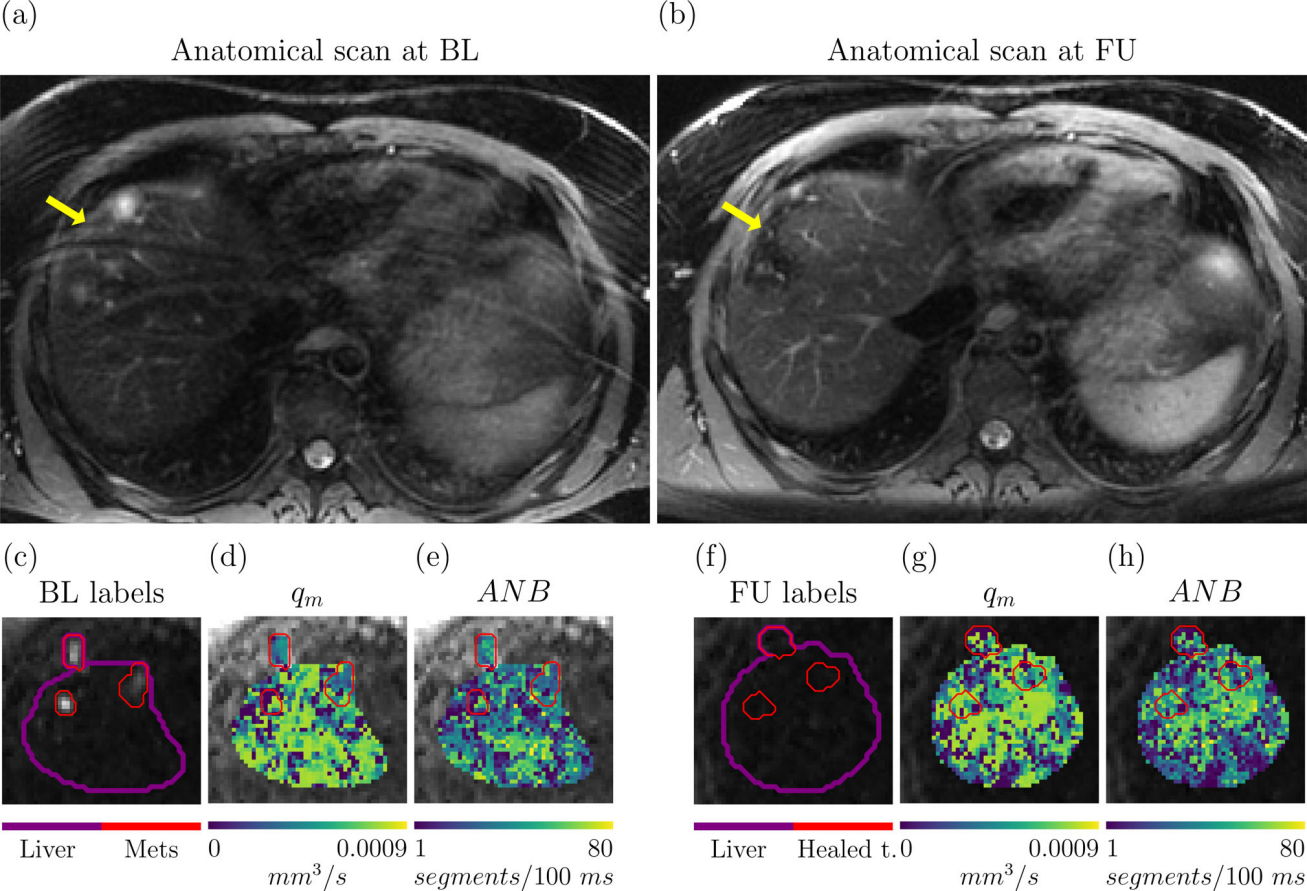
Microvascular parameter estimation in a CRC patient in vivo. Top: structural, anatomical high‐resolution T2‐weighted scan of the liver obtained at baseline (BL) and at follow‐up (FU), after 3 months of targeted therapy for metastatic CRC. (a) BL scan, with arrow illustrating the position of two metastases. (b) FU scan, highlighting the effect of treatment, with healed liver parenchyma replacing the metastases. Bottom: microvascular maps $q_{m}$ (mean VFR) and ANB (apparent network branching) at the two time points. From left to right, (c) high b‐value image highlighting the position of 3 CRC metastases within the liver at BL; (d) $q_{m}$ map at BL; (e) ANB map at BL; (f) high b‐value image at FU, in the same liver location featuring metastases at BL, now featuring healed tissue; (g) $q_{m}$ map at FU; (h) ANB map at FU.

Figure 8 shows MRI data from the metastatic CRC patient at baseline, and after 3 months of targeted therapy against liver metastases. The figure reveals the location of the metastases within the liver parenchyma, and demonstrates that these are replaced by healed parenchymal tissue after treatment. The figure also depicts $q_{m}$ and ANB maps. At baseline, on visual inspection both metrics appear lower in the metastases than in the liver. At follow‐up, both $q_{m}$ and ANB increases, recovering values similar to those of the liver parenchyma.

Table 1 reports mean and standard deviation of $q_{m}$, ANB, and of IVIM $f_{V}$ and $D^{*}$ in multiple ROIs. Quantitative trends confirm what was observed on visual inspection. The liver features higher vascularisation than both spleen and metastases (higher $f_{V}$, $D^{*}$, $q_{m}$, and ANB). After treatment, all vascular metrics in the healed metastases increase, recovering values that match closely those of the liver parenchyma ROI.

**TABLE 1:** Mean and standard deviation of metrics from simulation‐informed and IVIM parameter fitting in different ROIs. The table reports metrics for the colorectal cancer (CRC) patient and for the healthy volunteer. For the CRC case, results from both baseline (BL) and follow‐up (FU) scans are included.

ID	ROI	$q_{m}$ [$10^{-3}{\text{mm}}^{3}$/s]	ANB [segments/100 ms]	$f_{v}$	$D^{*}$ [μ $m^{2}$/ms]
Patient at BL
	Metastases	0.279 (0.170)	23.9 (15.3)	0.230 (0.217)	8.70 (16.5)
	Spleen	0.305 (0.283)	28.7 (20.6)	0.185 (0.213)	15.1 (38.6)
	Liver	0.539 (0.270)	38.3 (22.5)	0.598 (0.298)	18.7 (35.7)
Patient at FU
	Healed metastases	0.571 (0.229)	41.7 (19.7)	0.384 (0.335)	20.6 (38.8)
	Spleen	0.446 (0.290)	37.7 (20.9)	0.131 (0.178)	24.8 (48.9)
	Liver	0.565 (0.244)	38.8 (21.0)	0.455 (0.316)	23.0 (47.5)
Volunteer
	Spleen	0.439 (0.252)	39.9 (18.7)	0.123 (0.124)	23.7 (43.3)
	Liver	0.542 (0.215)	49.1 (16.6)	0.359 (0.227)	45.9 (62.9)

Table 1 also lists statistics in the healthy volunteer's spleen and liver ROI, which are in line with values seen in the patient (maps in Supporting Information: Figure S15).

## 4 Discussion

### 4.1 Summary

Using simulations of blood flow in capillary networks, we studied microvascular parameter mapping with diverse dMRI protocols. Our analysis shows that the directionally‐averaged signal encodes 2–3 key parameters at realistic SNR, primarily the mean volumetric flow rate ($q_{m}$) and a measure of network complexity (ANB). This number goes up to 10–50 without directional averaging. The feasibility of estimating these metrics in vivo was tested on a healthy volunteer and on a metastatic CRC patient with liver metastases. The metastases showed lower $q_{m}$ and ANB compared to non‐cancerous liver tissue, agreeing with standard IVIM. After three months of targeted therapy, these differences were reversed, demonstrating the potential of simulation‐informed microvascular measurement for monitoring treatment response.

### 4.2 In Silico Study

The estimation of the degrees of freedom, based on signal matrix SVD, demonstrates that at realistic SNR (e.g., between 5 to an optimistic upper bound of 20 [37] on the vascular signal), 2–3 independent components can be captured with directionally‐averaged signals. This number increases to 10 to 50, in the best SNR case of 20, without directional averaging—a finding that, to the best of our knowledge, is being reported here for the first time. These findings suggest that around 3 fully independent microvascular parameters can be practically estimated from clinical dMRI acquisitions, which typically use directionally‐averaged trace imaging. When richer sets of gradient directions are available instead, a higher number of significant SVs may be detected. Noting that the vascular pseudo‐diffusion tensor (first order cumulant) contains 6 independent parameters [38], this implies that higher‐order directional signal features (e.g., vascular kurtosis tensors [39, 40]) could be exploited for the estimation of tensorial extensions of the scalar metrics considered here. However, it should be remembered that care is needed when interpreting the pseudo‐diffusion and kurtosis tensors on the hybrid protocols, as these encompass both FC and NC measurements, each probing different time scales. While these tensors could be introduced for the FC and NC protocols individually, their direct biophysical meaning is difficult to pinpoint when a protocol contains both FC and NC measurements.

Afterwards, we investigated which microvascular properties could be estimated in practice from noisy signal measurements. We ranked properties using two criteria: one quantifying detectability and based on the correlation between ground truth and estimated parameters ($r_{s}$), and the other assessing property estimation accuracy through the BI, in absolute value. The detectability ranking suggests that for a variety of acquisition settings, the moments of the VFR distribution ($q_{m}$ and $q_{s}$) and the ANB are the metrics to which the signal is mostly sensitive. *For this reason*, $q_{m}$ *and* ANB *are the properties we recommend focussing estimation efforts on, when two metrics are of interest for in vivo microvascular mapping*. Interestingly, these results also suggest that the VFR distribution can be better recovered than the blood velocity distribution. This result, heretofore undescribed, shows the potential of physics‐informed simulations to guide the design of new MRI biomarkers.

Another important observation from the in silico study is that metrics related to the network geometry, as the mean capillary radius/length, cannot be recovered even at high SNR as they show very low detectability. This likely results from the fact that the VFR q and the velocity v are directly related to the spin trajectories p encoded in the spin phase ($v(t)=\frac{d}{dt}p(t)$; q=∫v(t)·dA, where dA is the elementary area element). Conversely, indices of capillary geometry alone (e.g., radius or length) are not sufficient to determine the overall flow resistance offered by a network, and hence the corresponding VFR field and the spin trajectories, unless information on the capillary arrangement (e.g., serial vs parallel [23]) is known.

The comparison between estimated and ground truth vascular parameters highlights other notable facts. For example, the choice of the diffusion time impacts detectability, with longer diffusion times yielding better performances for both NC and FC acquisitions. Moreover, hybrid acquisitions that combine both NC and FC measurements outperform FC and NC protocols. This is in line with known literature [16, 41, 42], since using both types of acquisition increases the contrast across DW measurements.

The second metric ranking performed in this study assessed the bias of the estimation, and hence quantifies accuracy. While metrics ranking high in detectability ($r_{s}$ criterion, that is, $q_{m}$ and ANB) also rank high in terms of bias at high SNR, results are more variable at the realistically low SNR level of 5. In this case, statistics of the blood velocity distribution such as $v_{m}$, while estimated more variably, show considerably less bias than $q_{m}$. This finding shows that sensitivity of the dMRI signal towards a metric (i.e., the metric detectability), does not necessarily supports accurate estimation. This observation, jointly with the moderate correlation values observed between ground truth and estimated tissue parameters (around 0.65), implies that while it seems feasible to characterise general trends in terms of $q_{m}$ and ANB contrast across tissues, further work is required to enable the accurate and precise measurement of these metrics in real world clinical settings.

We also characterised salient features of the vascular dMRI signal across multiple protocols. This analysis reveals a clear non‐mono‐exponential behaviour of the signal as a function of b (non‐zero kurtosis). It highlights diffusion‐time dependence, in agreement with previous studies [23], and shows that both vascular pseudo‐diffusion and kurtosis coefficients ($D^{*}$ and $K^{*}$) are sensitive to $q_{m}$ and ANB. This result stresses the importance of adequate b‐value sampling design for robust microvascular parameter mapping, in order to capture not only the slope of the (log)‐signal decay ($D^{*}$), but also changes in curvature ($K^{*}$).

Lastly, further analyses show that while a range of ANB values can be observed for any $q_{m}$ (Supporting Information: Figure S14), the two metrics are positively correlated. This suggests that additional vascular parameters could be potentially inferred jointly with $q_{m}$ and ANB, given that 2–3 *fully independent* degrees of freedom appear encoded in the signal.

### 4.3 In Vivo Study

We also tested whether $q_{m}$ and ANB could be estimated at 3T in vivo on a healthy volunteer and on a CRC patient, treated with targeted therapy against liver metastases. Results point towards the feasibility of estimating these metrics in vivo. While ANB mapping was previously demonstrated in our recent study [23], here we provide further evidence towards its robustness across protocols. Notably, the values of ANB reported here (between 20–50 segments) are higher than the number of capillaries through which spins have to travel in order to transition from ballistic to diffusive flow (around 7 at clinical diffusion times [4]). This finding can be explained by the fact that ANB is calculated over a reference time of 100 ms, hence considerably longer than standard diffusion times. For characteristic capillary segment lengths and blood velocities [23] of 0.060 mm and 3 mm/s, spins travel through 7 segments in around 20 ms. Conversely, they flow through around 35 segments in 100 ms—a figure in line with our ANB values.

To our knowledge this is the first time that VFR has been mapped in vivo with dMRI. Both ANB and VFR show trends that are plausible, highlighting temporal changes potentially associated with treatment effects, and thus demonstrating the potential of physics‐informed microvascular dMRI modelling for response assessment in oncology.

### 4.4 Methodological Considerations and Limitations

Firstly, we acknowledge that further confirmation is required from the simulation of larger and more complex networks, obtained, for example, through generative approaches [43] or from 3D histology [20, 44].

Secondly, in our in silico experiments we analysed signals with different b‐value samplings, and performed metric ranking on multiple diffusion times. This was done to ensure that results were not specific to a single acquisition, striving for generalisability. However, we acknowledge that further work is required: (i) to fully characterise the impact of Δ, δ, G, and τ on vascular metric estimation; (ii) to design acquisition protocols that maximise the signal sensitivity to a target metric of interest, for example, through Cramer‐Rao Lower Bound optimisation [45]; (iii) to assess the estimation of tensorial extensions of the vascular metrics considered here.

Moreover, in our simulations we used well‐established SV decomposition to investigate the number of recoverable, independent vascular components. Given the non‐Gaussian nature of the noise injected on the synthetic signals, it is possible that at least one of the significant signal components $N_{p}$ is capturing the Rician noise floor. Moreover, care is needed to generalise such findings obtained in silico to in vivo data, where noise is likely tobe heteroscedastic (e.g., due to a varying number of signal averages across b‐values).

Another important point to consider is related to the approach used to learn numerical signal models. Here we used RBF regressors, since these are light, versatile models that can be used to interpolate discrete parameter‐signal dictionaries [23]. Nonetheless, we acknowledge that other promising approaches are also possible, as for example those based on regression‐kriging, Gaussian processes or machine/deep learning. We aim to explore these in future work.

Regarding the dMRI protocols, our simulations highlighted the benefits of combining FC and NC measurements. The use of FC waveforms may be especially beneficial in vivo, since these compensate for some of the unavoidable patient's bulk motion [41]. However, it should be remembered that the FC waveforms may suffer from reduced diffusion‐weighting efficiency, and decreases in SNR [46]. Moreover, the intrinsic slower decay rate of FC vascular signals as a function of b, compared to NC measurements, implies that care would be needed when using segmented fitting on FC acquisitions. This is due to the fact that residual vascular contributions may coexist with extra‐vascular signals even at b‐values well above 100–250 s/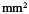.

Lastly, we acknowledge that further validation in larger patient data bases is required.

### 4.5 Conclusions

Physics‐based simulations of the vascular dMRI signal inform the design of innovative microperfusion biomarkers for in vivo imaging. The estimation of two to three fully independent vascular parameters from IVIM‐like acquisitions appears feasible in vivo, with VFR distribution moments and indices of vascular network branching being the most promising metrics to capture salient trends in microcapillary perfusion. While further work is needed to boost the accuracy and precision of the estimation of these metrics for real‐world deployment, our study highlights the potential of non‐invasive, quantitative microvascular dMRI for personalised imaging in oncology.

## Funding

This work was supported by the Agencia de Gestio d'Ajuts Universitaris i de Recerca (2023PROD00178, SGR‐Cat2021), the Fundacion Cientifica Asociacion Espanola Contra el Cancer (PRYCO211023SERR), the UK Research and Innovation (MR/T020296/2), the CRIS Cancer Foundation (TALENT19‐05), the Prostate Cancer Foundation (18YOUN19), the FERO Foundation, the Instituto de Salud Carlos III (PI18/01395), the Agencia Estatal de Investigacion (CEX2020‐001024‐S/AEI/10.13039/501100011033, PRE2022‐102586), the 'la Caixa' Foundation : CaixaResearch Advanced Oncology Research Program (LCF/BQ/PR22/11920010), the Fundacion BBVA (89/2017), the Cellex Foundation, the Fundació Institució dels Centres de Recerca de Catalunya (CERCA), and the European Regional Development Fund (European Commission, EU)

## Conflicts of Interest

The authors declare no conflicts of interest.

## Supporting information




**Data S1:** Supporting information.

## Data Availability

The simulations performed in this study are based on the SpinFlowSim perfusion simulator and on the vascular networks released freely with the simulator (permanent address: https://github.com/radiomicsgroup/SpinFlowSim). The 3D networks and the code generating the perturbations will be released upon publication at the permanent address: https://github.com/radiomicsgroup/SpinFlowSim/tree/main/networks3D. The in vivo MRI data cannot be made freely available at this stage due to ethical considerations. Researchers interested in accessing the data can contact the corresponding author, so that appropriate institutional data transfer agreements can be stipulated.
