# Supplementary material for: Simulation‐Informed Evaluation of Microvascular Parameter Mapping for Diffusion MR Imaging of Solid Tumours
Source: Magn Reson Med. 2026 Mar 7;96(1):387–402. doi: 10.1002/mrm.70318 (PMC13156458; doi:10.1002/mrm.70318)
Supplement: Supplementary file 1 — Data S1: Supporting information. [file MRM-96-387-s001.pdf]

Supporting Information

Supporting Information Table S1. Number of independent vascular components N_p above the noise level detected on the noisy synthetic signal by hard thresholding of the singular values (SVs). The table reports N_p as the hard threshold θ varies from 0.07 to 0.80. In practice, a hard threshold equal to θ implies that the signal carried by a SV is considered detectable if the noisy version of the SV is at most $100\% \times \theta$ higher than the corresponding noise-free SV, in percentage terms. As an example, for $\theta = 0.21$, the signal carried by a SV is considered detectable if the SV amplitude is at most 21 % higher than that of the corresponding noise-free SV. Note that injecting noise on the synthetic signal effectively increases the numerical amplitude of the SVs. The table reports results for all protocols shown in Fig. 3.

**Protocol**	**SNR**	$\theta =$ 0.07	$\theta =$ 0.11	$\theta =$ 0.13	$\theta =$ 0.15	$\theta =$ 0.17	$\theta =$ 0.19	$\theta =$ 0.21	$\theta =$ 0.23	$\theta =$ 0.25	$\theta =$ 0.30	$\theta =$ 0.40	$\theta =$ 0.50	$\theta =$ 0.60	$\theta =$ 0.70	$\theta =$ 0.80
NC	5	2	2	2	2	2	2	2	2	2	2	2	2	2	2	2
$\Delta = 30$ ms, $\delta = 6$ ms	20	2	2	2	2	3	3	3	3	3	3	3	3	3	3	3
FC	5	2	2	2	2	2	2	2	2	2	2	2	2	2	2	2
$\Delta = 30$ ms, $\tau = 10$ ms	20	2	2	2	2	3	3	3	3	3	3	3	3	3	3	3
richNC, directional averaging	5	1	2	2	2	2	2	2	2	2	2	2	2	2	2	2
$\Delta = 30$ ms, $\delta = 6$ ms	20	2	3	3	3	3	3	3	3	3	3	4	4	4	4	4
richFC, directional averaging	5	1	2	2	2	2	2	2	2	2	2	2	2	2	2	2
$\Delta = 30$ ms, $\tau = 10$ ms	20	2	3	3	3	3	3	3	3	3	3	3	4	4	4	4

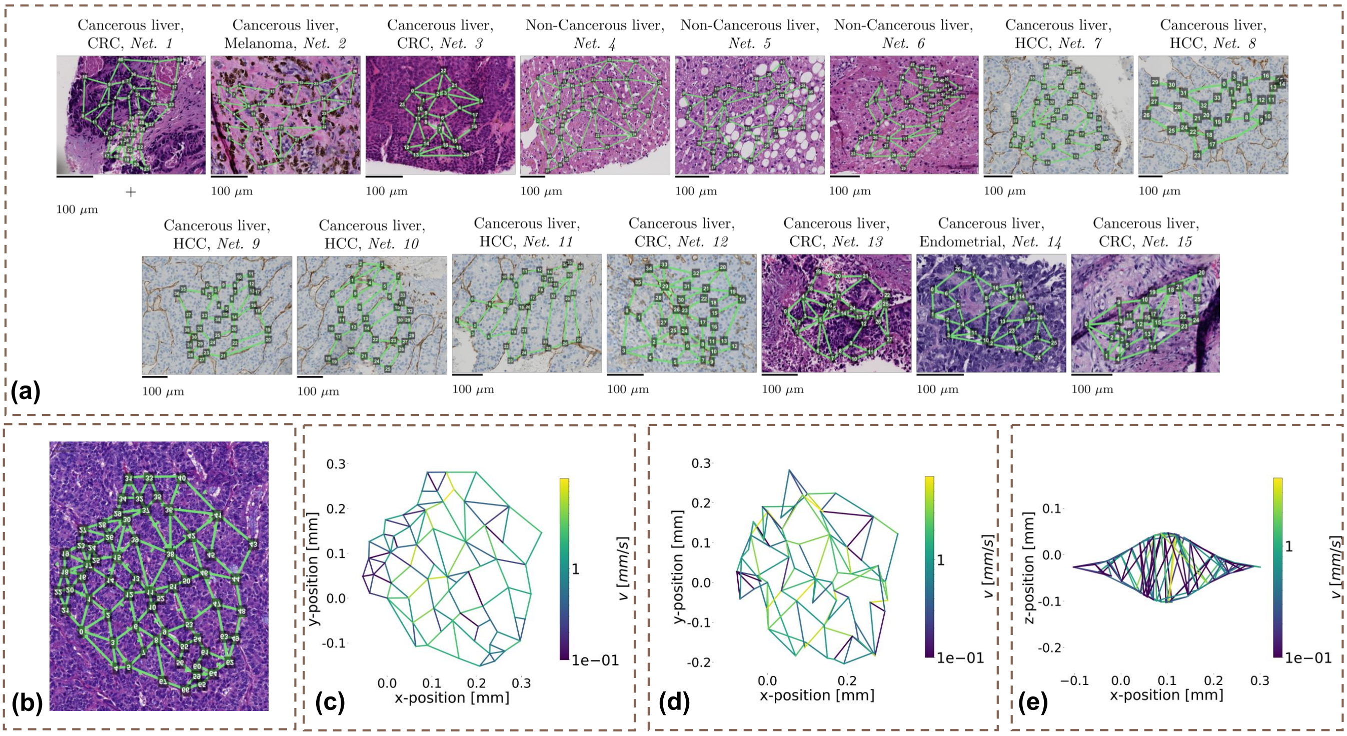

Supporting Information Figure S1 Illustration of the vascular networks used in our article. (a): the 15 2D freely available networks from a previous study[23] processed for our experiments *in silico*. (b)-(d): illustration of the perturbation procedure followed to generate a rich set of 1500 tree-dimensional and unique networks. (b): 2D network; (c): 2D network with resolved velocity field; (d): the same network after perturbation of radii and node positions; (e): final 3D shape of the network. Panel (a) has been generated with permission by adapting Figure 2 of Voronova et al[23], Medical Image Analysis 2025, 102: 103531, doi: 10.1016/j.media.2025.103531. Journal: Medical Image Analysis, Elsevier (https://www.sciencedirect.com/journal/medical-image-analysis). License number 6078220596843 obtained on July 29th 2025 through CCC RightsLink.

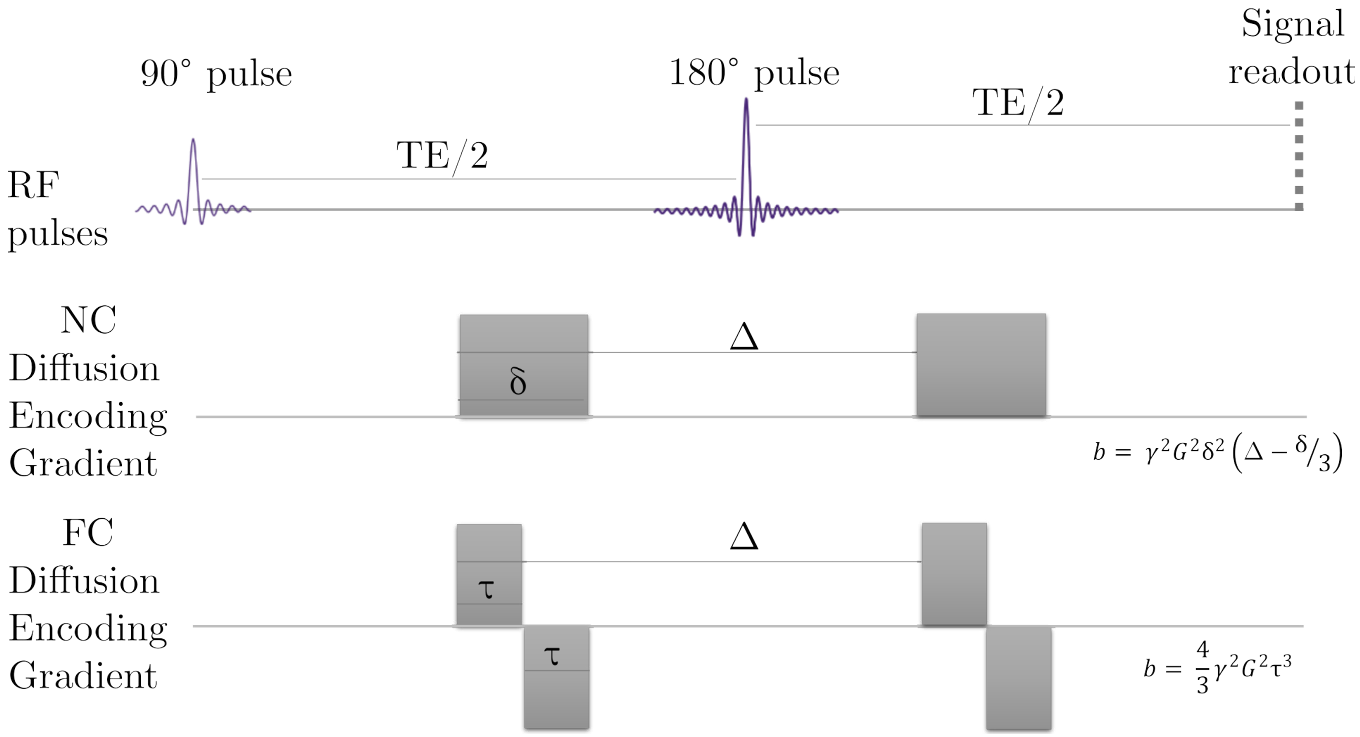

Supporting Information Figure S2 Illustration of the NC and FC gradient waveforms used in this study, with corresponding b-value expressions.

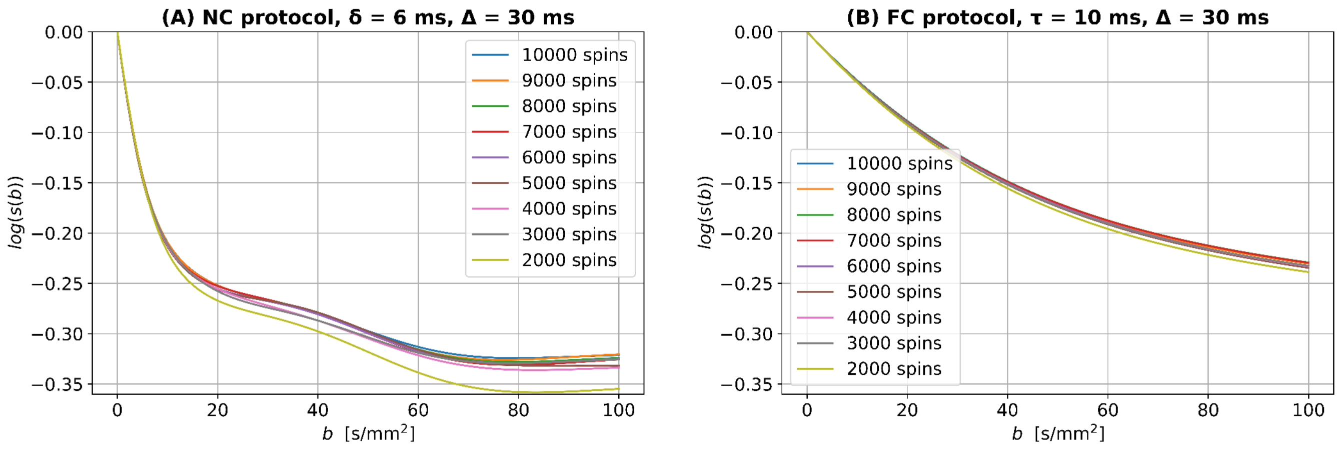

Supporting Information Figure S3 Example of the effect that the number of spins used for simulating capillary flow has on the signal synthesis. (A): results for a NC protocol featuring $\delta = 6$ ms and $\Delta = 30$ ms. (B): results for a FC protocol featuring $\tau = 10$ ms and $\Delta = 30$ ms. The signals were obtained by averaging over 9 uniformly distributed gradient directions. Signals correspond to one realisation of network 7. The plots report the decimal logarithm of the signal against the b-value b.

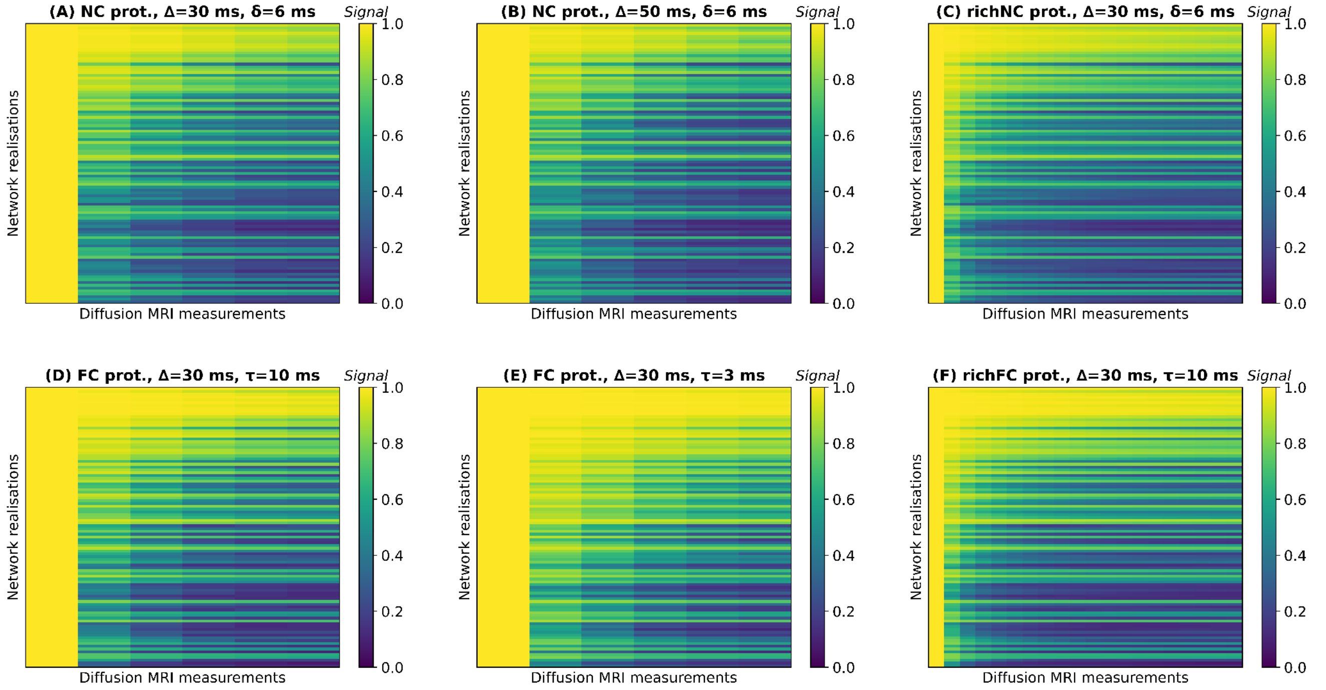

Supporting Information Figure S4 Visualisation of all 100 noise-free synthetic signals generated for network 7. Each panel visualises the magnitude of the synthesised diffusion-weighted signal. Different diffusion-weighted measurements are stacked along the columns (horizontal axis), while the 100 unique network realisations are stacked along the rows (vertical axis). The figure illustrates different protocols with directional averaging. (A): NC protocol, $\Delta = 30$ ms, $\delta = 6$ ms. (B): NC protocol, $\Delta = 50$ ms, $\delta = 6$ ms. (C): richNC protocol, $\Delta = 30$ ms, $\delta = 6$ ms. (D): FC protocol, $\Delta = 30$ ms, $\tau = 10$ ms. (E): FC protocol, $\Delta = 30$ ms, $\tau = 3$ ms. (F): richFC protocol, $\Delta = 30$ ms, $\tau = 10$ ms.

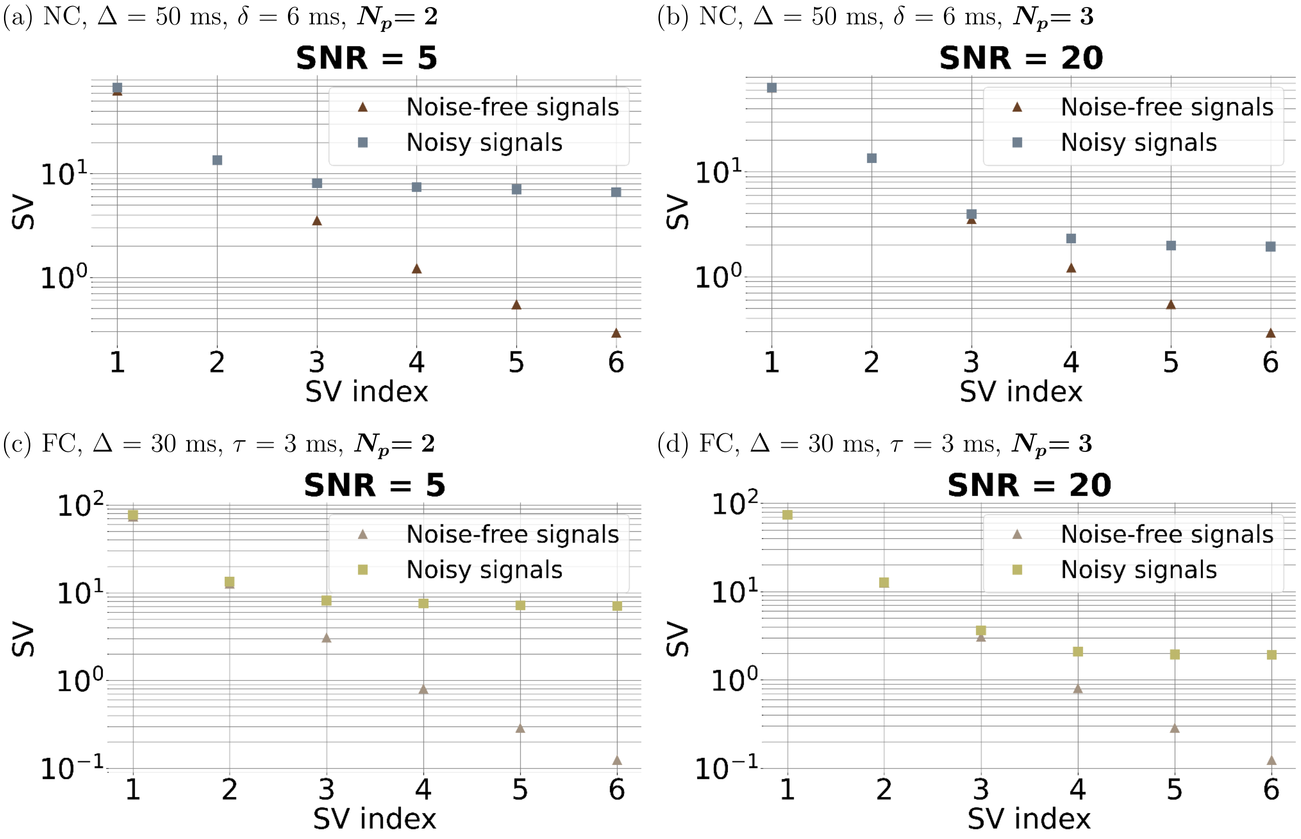

Supporting Information Figure S5 SVD for the non-compensated (NC) and flow-compensated (FC) protocols following directional averaging for a second diffusion time. Left, panels (a), (c): vascular dMRI signal for SNR of 5 at $b = 0$; right, panels (b), (d): vascular dMRI signal for SNR of 20 at $b = 0$. From top to bottom: NC protocol ((a) and (b)); FC protocol ((c) and (d)). The figure refers to the fixed diffusion time of $\delta = 6$ ms and $\Delta = 50$ ms (NC protocol), and of $\tau = 3$ ms and $\Delta = 30$ ms (FC protocol).

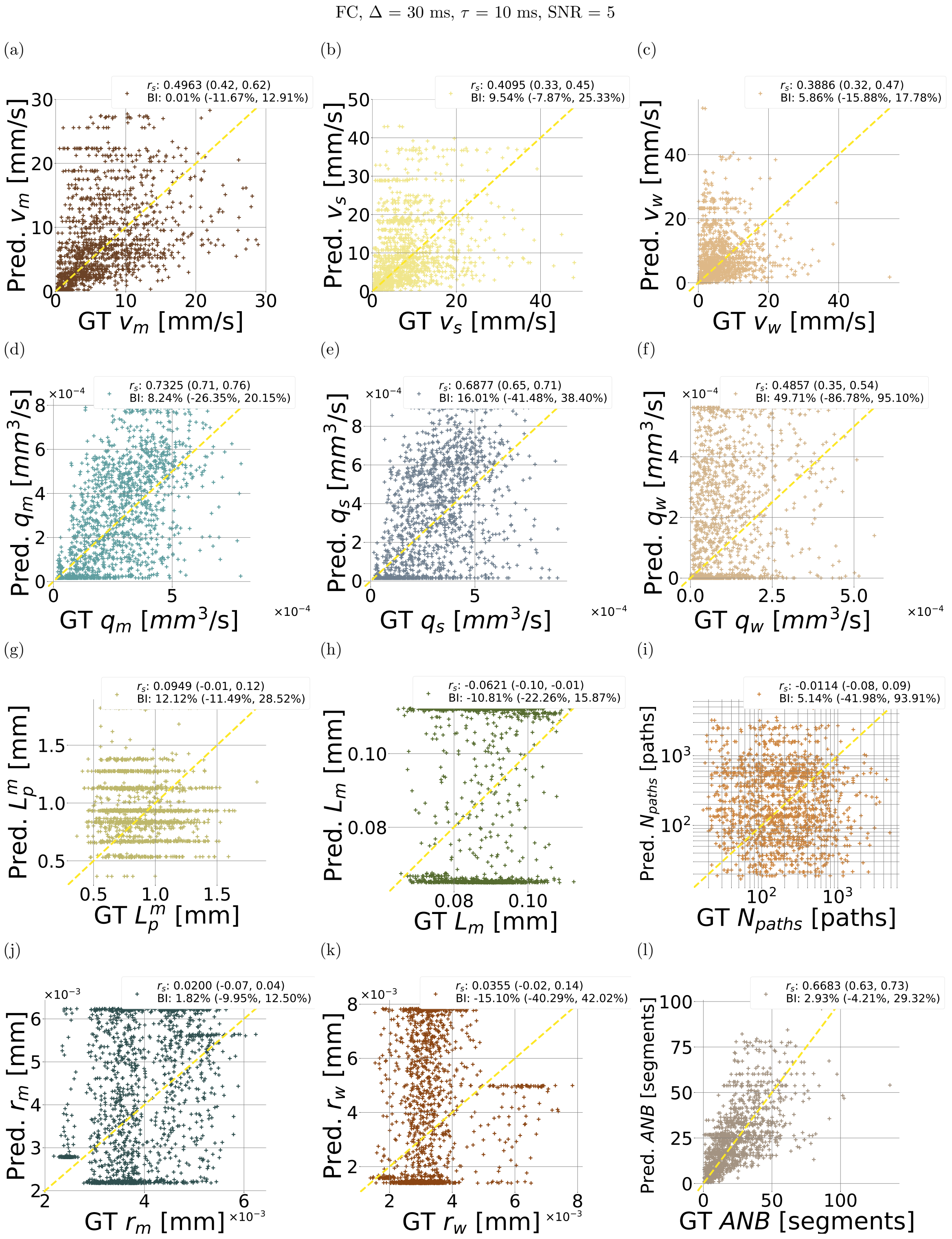

Supporting Information Figure S6 Scatter plots of estimated vascular parameters against ground truth values from the leave-one-out fitting procedure implemented *in silico*. The figure refers to the FC protocol, with $\Delta = 30$ ms and $\tau = 10$ ms, SNR $= 5$. From top to bottom: first row, mean velocity v_m in (a), standard deviation of velocity v_s in (b), path-weighted mean velocity v_w in (c); second row, mean volumetric flow rate (VFR) q_m in (d), standard deviation of VFR q_s in (e), path-weighted mean VFR q_w in (f); third row, mean input/output path length L_p^m in (g), mean capillary segment length L_m in (h), number of input/output paths N_{paths} in (i); fourth row, mean capillary radius r_m in (j), path-weighted mean capillary radius r_w in (k), and apparent network branching ANB in (l). For each metric, the overall Spearman's correlation coefficient r_s and Bias Index (BI) are reported, with the range of r_s and BI values obtained across leave-one-out folds. . "GT" and "Pred." respectively indicate ground truth and predicted metric values.

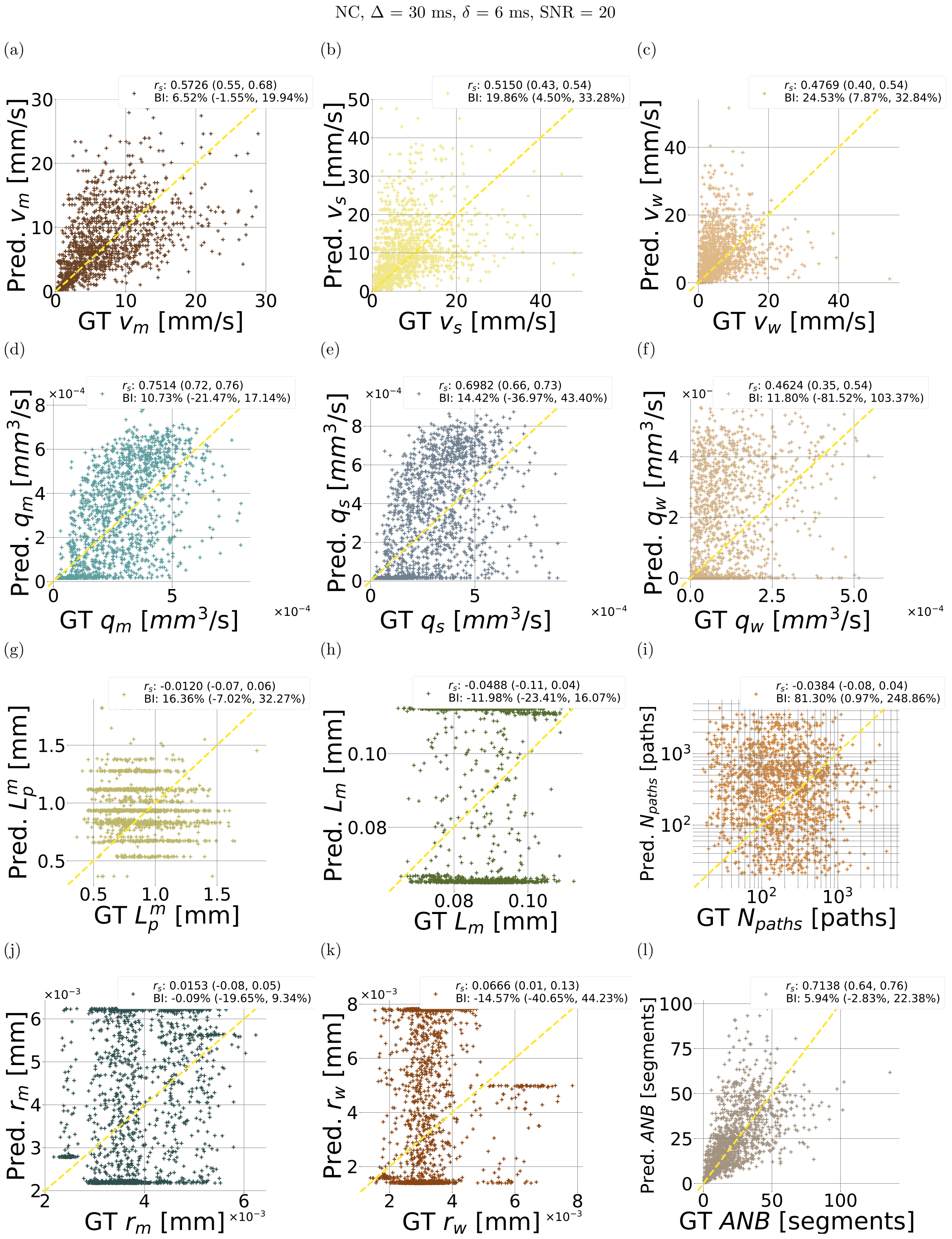

Supporting Information Figure S7 Scatter plots of estimated vascular parameters against ground truth values from the leave-one-out fitting procedure implemented *in silico*. The figure refers to the NC protocol, with $\Delta = 30$ ms and $\delta = 6$ ms, SNR $= 20$. From top to bottom: first row, mean velocity v_m in (a), standard deviation of velocity v_s in (b), path-weighted mean velocity v_w in (c); second row, mean volumetric flow rate (VFR) q_m in (d), standard deviation of VFR q_s in (e), path-weighted mean VFR q_w in (f); third row, mean input/output path length L_p^m in (g), mean capillary segment length L_m in (h), number of input/output paths N_{paths} in (i); fourth row, mean capillary radius r_m in (j), path-weighted mean capillary radius r_w in (k), and apparent network branching ANB in (l). For each metric, the overall Spearman's correlation coefficient r_s and Bias Index (BI) are reported, with the range of r_s and BI values obtained across leave-one-out folds. “GT” and “Pred.” respectively indicate ground truth and predicted metric values.

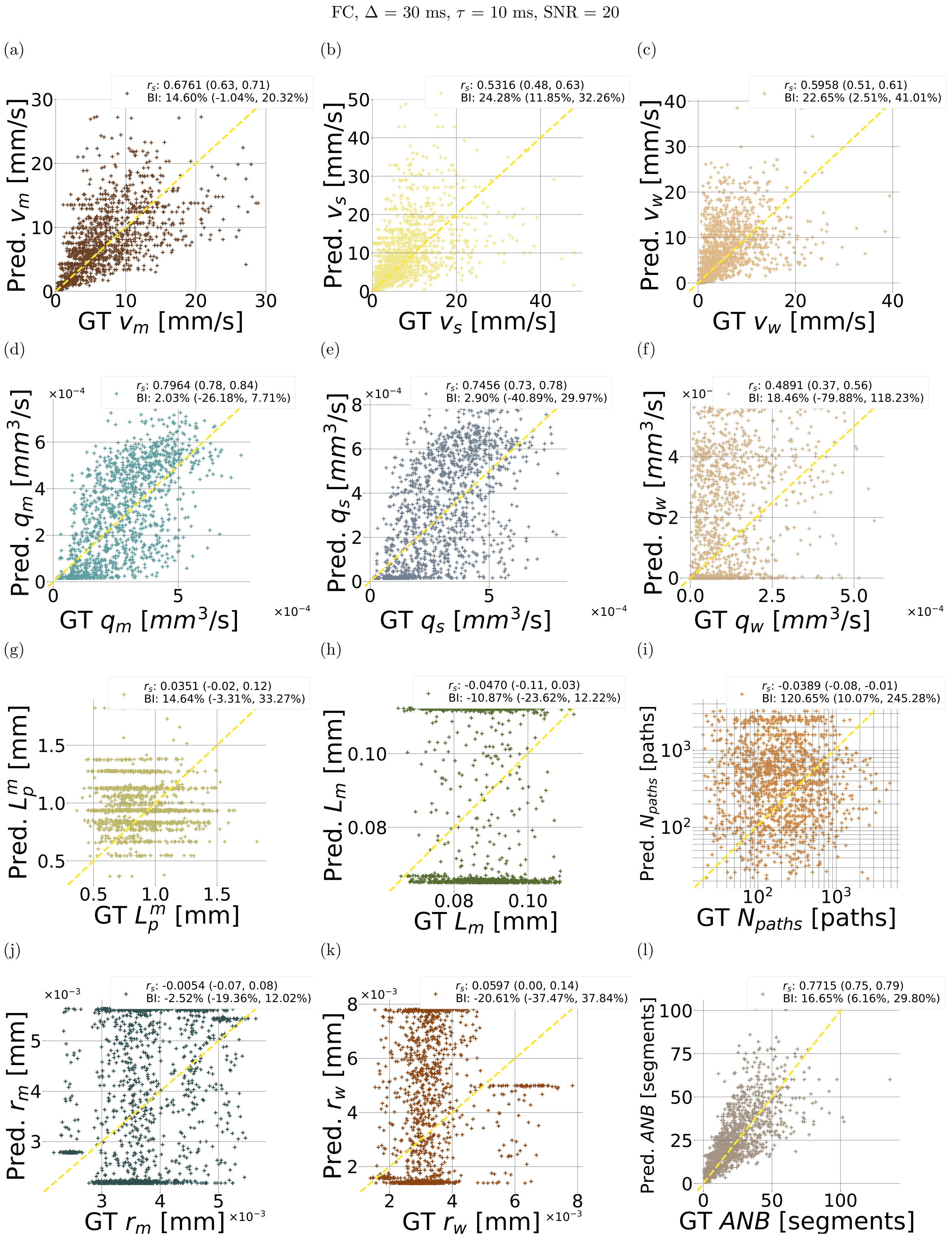

Supporting Information Figure S8 Scatter plots of estimated vascular parameters against ground truth values from the leave-one-out fitting procedure implemented *in silico*. The figure refers to the FC protocol, with $\Delta = 30$ ms and $\tau = 10$ ms, SNR $= 20$. From top to bottom: first row, mean velocity v_m in (a), standard deviation of velocity v_s in (b), path-weighted mean velocity v_w in (c); second row, mean volumetric flow rate (VFR) q_m in (d), standard deviation of VFR q_s in (e), path-weighted mean VFR q_w in (f); third row, mean input/output path length L_p^m in (g), mean capillary segment length L_m in (h), number of input/output paths N_{paths} in (i); fourth row, mean capillary radius r_m in (j), path-weighted mean capillary radius r_w in (k), and apparent network branching ANB in (l). For each metric, the overall Spearman's correlation coefficient r_s and Bias Index (BI) are reported, with the range of r_s and BI values obtained across leave-one-out folds. "GT" and "Pred." respectively indicate ground truth and predicted metric values.

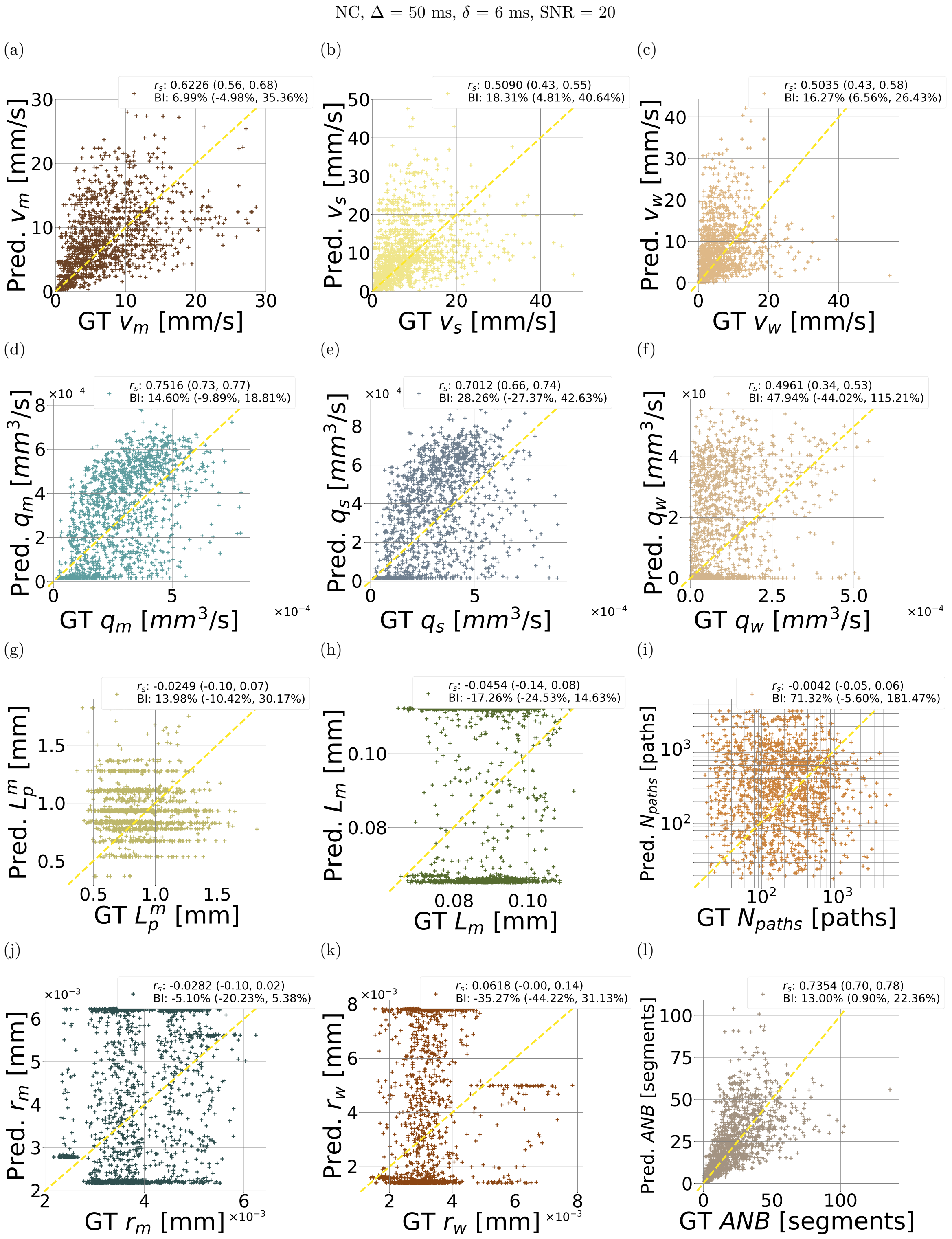

Supporting Information Figure S9 Scatter plots of estimated vascular parameters against ground truth values from the leave-one-out fitting procedure implemented *in silico*. The figure refers to the NC protocol, with $\Delta = 50$ ms and $\delta = 6$ ms, SNR = 20. From top to bottom: first row, mean velocity v_m in (a), standard deviation of velocity v_s in (b), path-weighted mean velocity v_w in (c); second row, mean volumetric flow rate (VFR) q_m in (d), standard deviation of VFR q_s in (e), path-weighted mean VFR q_w in (f); third row, mean input/output path length L_p^m in (g), mean capillary segment length L_m in (h), number of input/output paths N_{paths} in (i); fourth row, mean capillary radius r_m in (j), path-weighted mean capillary radius r_w in (k), and apparent network branching ANB in (l). For each metric, the overall Spearman's correlation coefficient r_s and Bias Index (BI) are reported, with the range of r_s and BI values obtained across leave-one-out folds. "GT" and "Pred." respectively indicate ground truth and predicted metric values.

FC, Δ = 30 ms, τ = 3 ms, SNR = 20

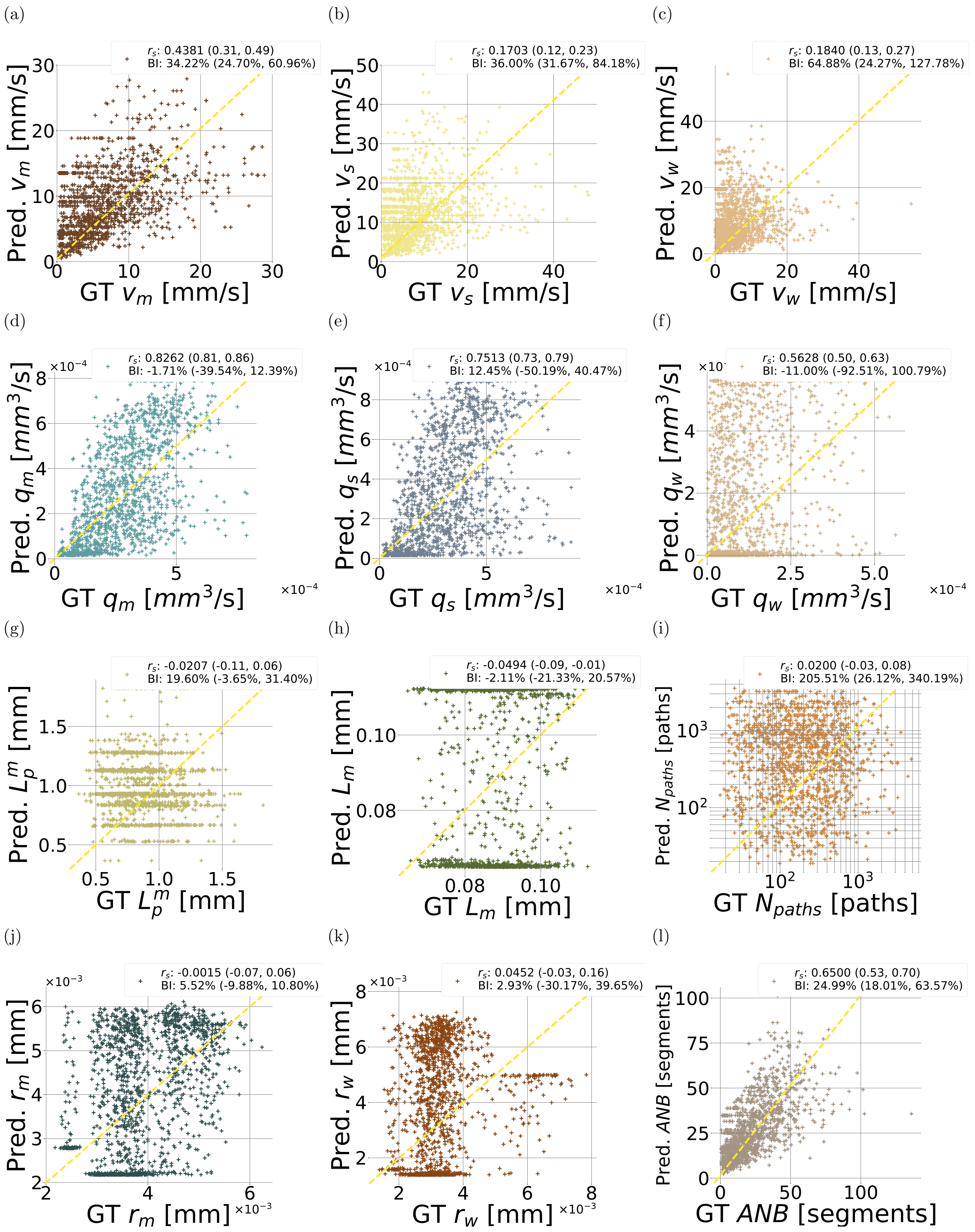

Supporting Information Figure S10 Scatter plots of estimated vascular parameters against ground truth values from the leave-one-out fitting procedure implemented *in silico*. The figure refers to the FC protocol, with Δ = 30 ms and τ = 3 ms, SNR = 20. From top to bottom: first row, mean velocity v_m in (a), standard deviation of velocity v_s in (b), path-weighted mean velocity v_w in (c); second row, mean volumetric flow rate (VFR) q_m in (d), standard deviation of VFR q_s in (e), path-weighted mean VFR q_w in (f); third row, mean input/output path length L_p^m in (g), mean capillary segment length L_m in (h), number of input/output paths N_{paths} in (i); fourth row, mean capillary radius r_m in (j), path-weighted mean capillary radius r_w in (k), and apparent network branching ANB in (l). For each metric, the overall Spearman's correlation coefficient r_s and Bias Index (BI) are reported, with the range of r_s and BI values obtained across leave-one-out folds. "GT" and "Pred." respectively indicate ground truth and predicted metric values.

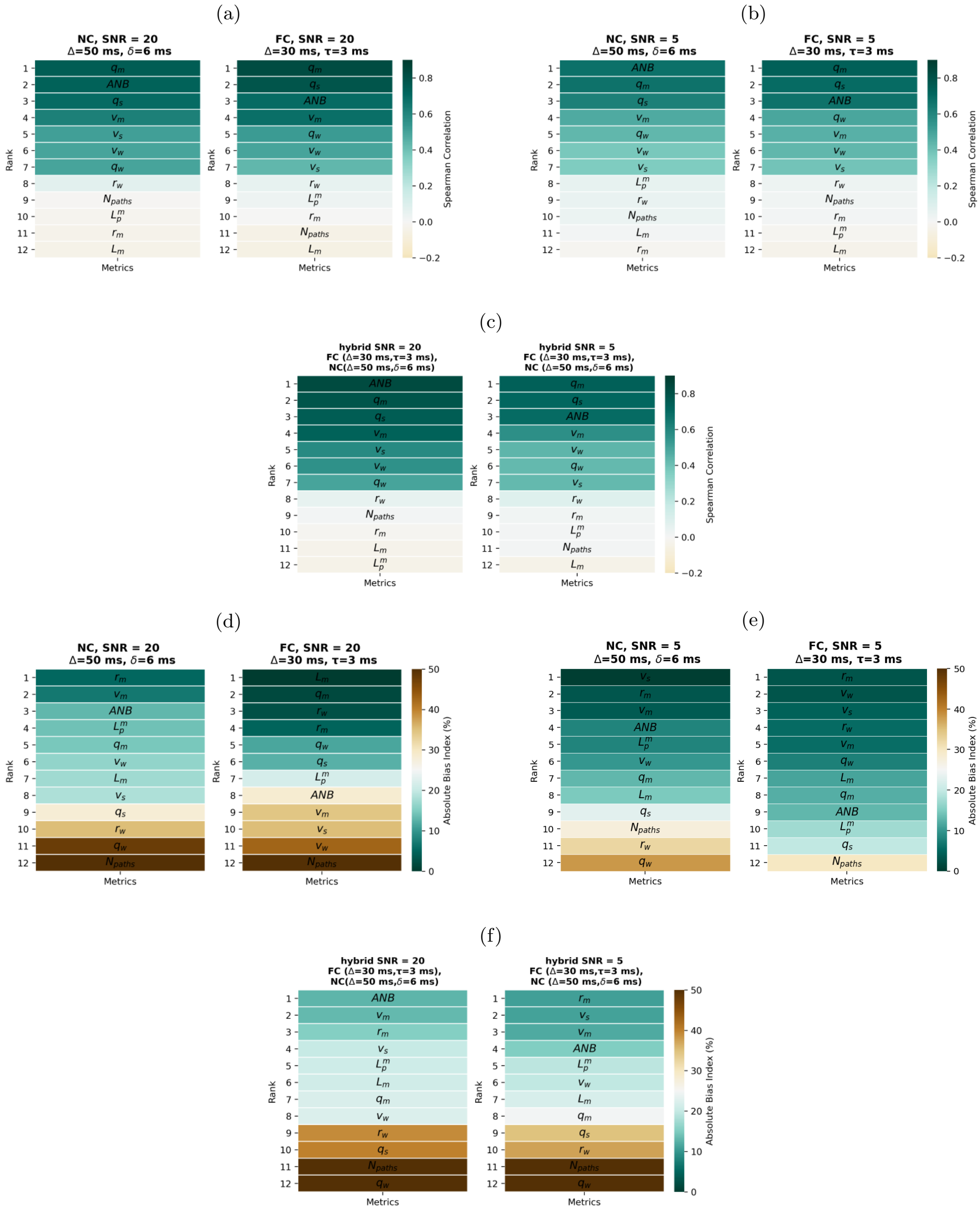

Supporting Information Figure S11 Ranking of microvascular metrics based on the Spearman's correlation r_s and on the bias index BI. (a): r_s ranking for the NC and FC protocols (SNR = 20); (b): r_s ranking for the NC and FC protocols (SNR = 5); (c): r_s ranking for the hybrid protocol (SNR = 20 and SNR = 5); (d): BI ranking for the NC and FC protocols (SNR = 20); (e): BI ranking for the NC and FC protocols (SNR = 5); (f): BI ranking for the hybrid protocol (SNR = 20 and SNR = 5). The figure refers to the following diffusion time: $\Delta = 30$ ms, $\delta = 6$ ms for the NC and hybrid protocol; $\Delta = 30$ ms, $\tau = 10$ ms for the FC and hybrid protocol.

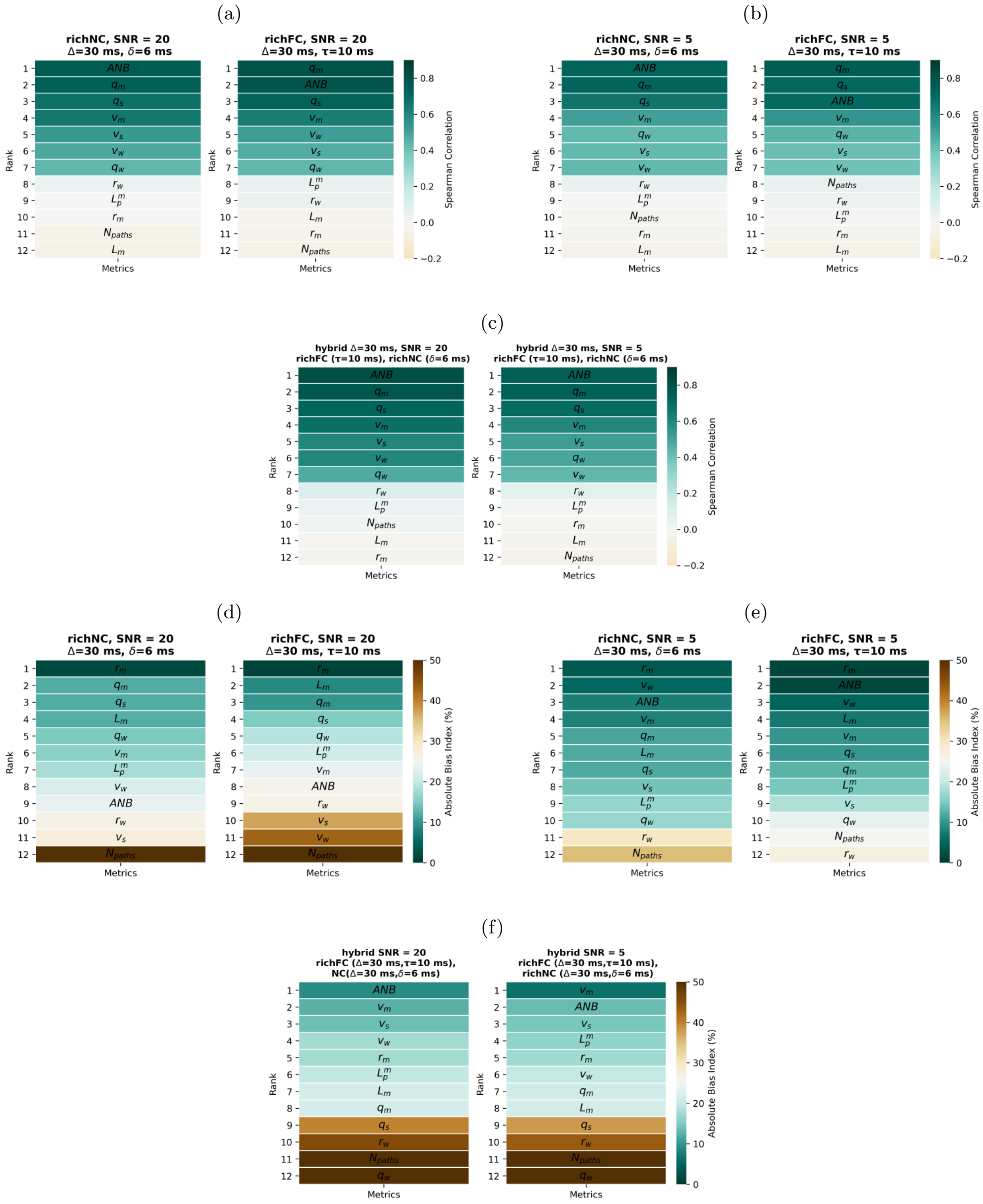

Supporting Information Figure S12 Ranking of microvascular metrics based on the Spearman's correlation r_s and on the bias index BI. (a): r_s ranking for the richNC and richFC protocols (SNR = 20); (b): r_s ranking for the richNC and richFC protocols (SNR = 5); (c): r_s ranking for the rich hybrid protocol (SNR = 20 and SNR = 5); (d): BI ranking for the richNC and richFC protocols (SNR = 20); (e): BI ranking for the richNC and richFC protocols (SNR = 5); (f): BI ranking for the rich hybrid protocol (SNR = 20 and SNR = 5). The figure refers to the following diffusion time: $\Delta = 30$ ms, $\delta = 6$ ms for the richNC and rich hybrid protocol; $\Delta = 30$ ms, $\tau = 10$ ms for the richFC and rich hybrid protocol.

FC protocol, Δ = 30 ms, τ = 10 ms, SNR = 20

(a) (b)

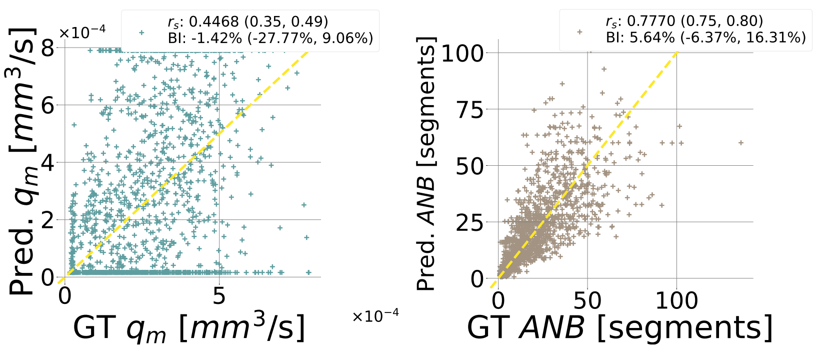

Supporting Information Figure S13 Example of quality of microvascular parameter fitting when two parameters are estimated jointly. The figure refers to the joint estimation of q_m and ANB for the flow-compensated (FC) protocol characterised by diffusion gradient timings of $\Delta = 30$ ms and $\tau = 10$ ms, for an SNR at $b = 0$ of 20.

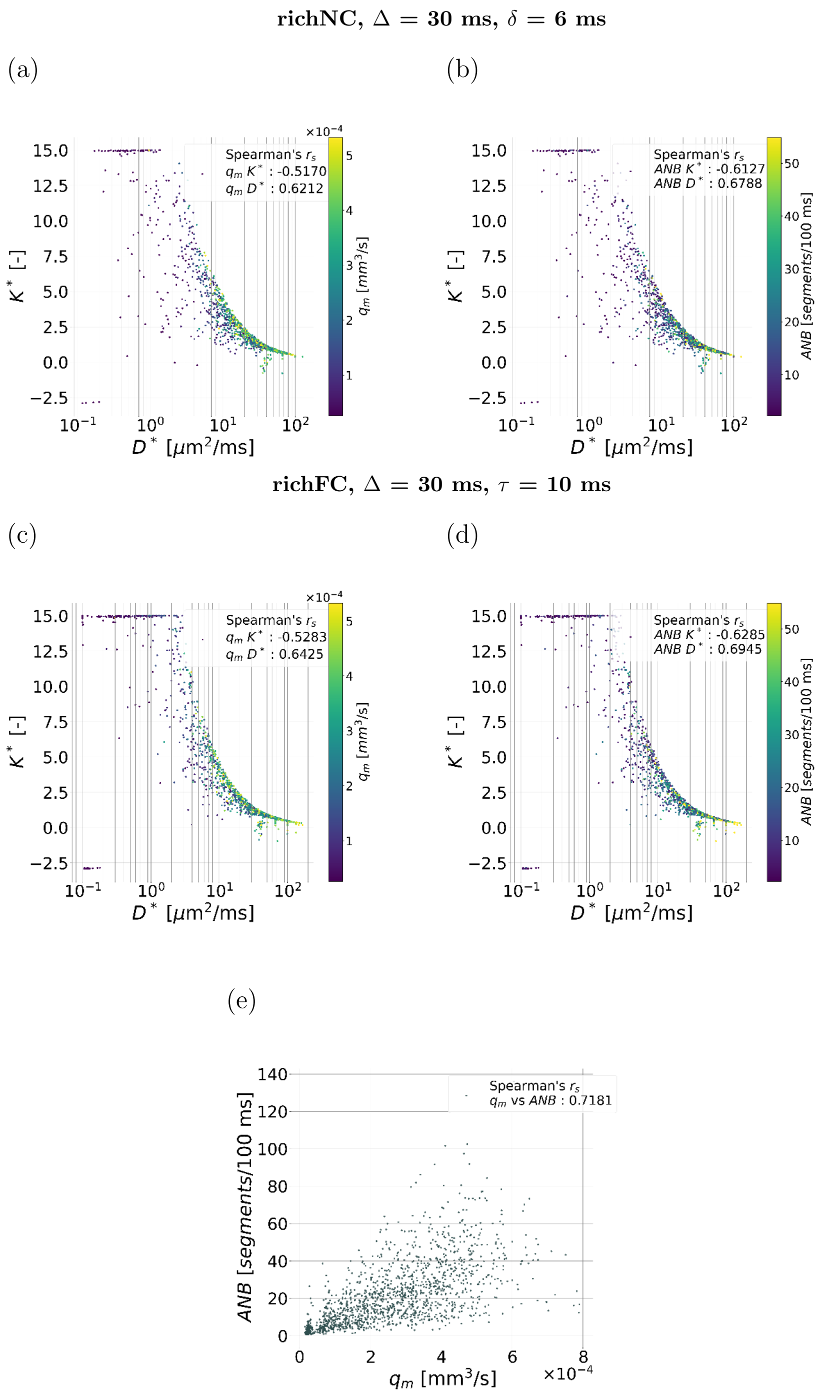

Supporting Information Figure S14 Scatter plots visualising the relationship between the vascular dMR signal cumulants and the top-ranking metrics selected from the *in silico* study. The figure scatters the apparent vascular diffusion and kurtosis coefficients (D^* and K^*) against each other, colouring the points according to the mean VFR q_m and the apparent network branching ANB, thus visualising the dependence of these two metrics on D^* and K^*, i.e., $q_m = f(D^*, K^*)$ and $ANB = f(D^*, K^*)$. Panels (a) and (b), top row: results for the richNC protocol ($q_m = f(D^*, K^*)$ in (a); $ANB = f(D^*, K^*)$ in (b); Spearman's correlation coefficients between q_m and D^* and K^*, and between ANB and D^* and K^* are also reported). Panels (c) and (d), central row: results for the richFC protocol ($q_m = f(D^*, K^*)$ in (c); $ANB = f(D^*, K^*)$ in (d); Spearman's correlation coefficients between q_m and D^* and K^*, and between ANB and D^* and K^* are also reported). Panel (e), bottom row: scatter plot relating q_m and ANB, with corresponding Spearman's correlation coefficient.

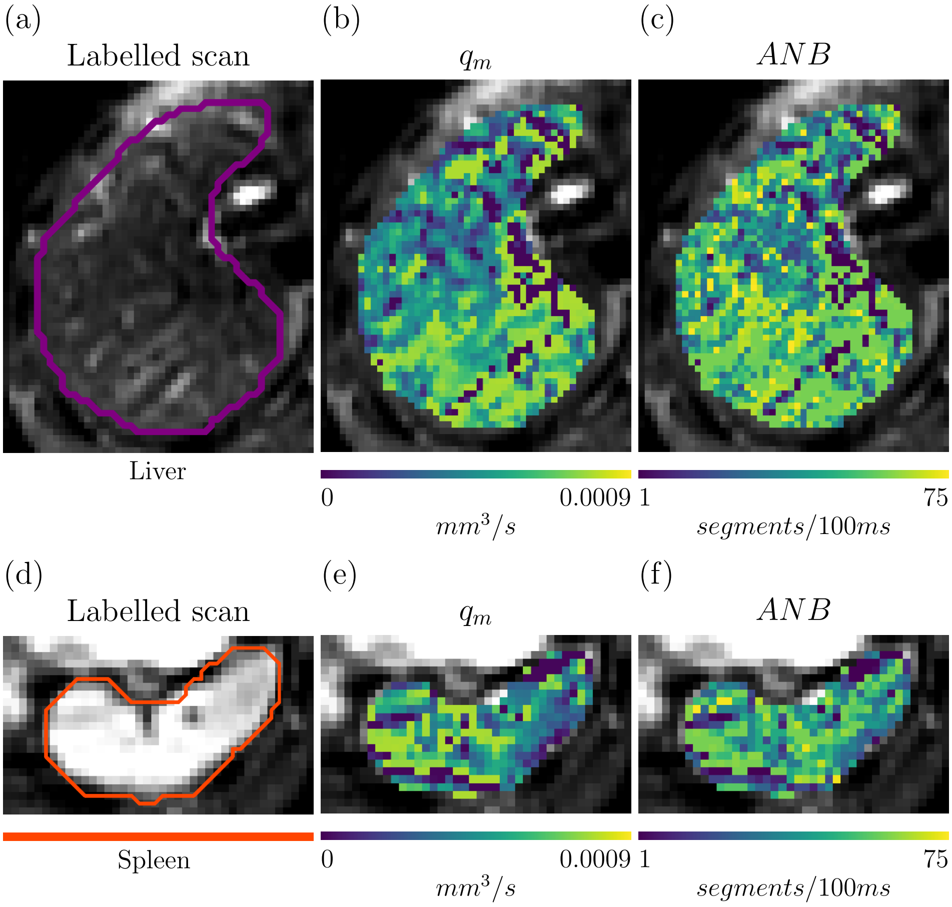

Supporting Information Figure S15 Microvascular parameter estimation in a healthy male volunteer *in vivo*. Top: microvascular parameter mapping in the liver parenchyma. From left to right, (a): mean $b = 0$ image of the liver; (b) q_m map in the liver; (c): ANB map in the liver. Bottom: microvascular parameter mapping in the spleen. From left to right, (d): mean $b = 0$ image of the spleen; (e) q_m map in the spleen; (f): ANB map in the spleen.
